# PH-Dependent Enantioselectivity of D-amino Acid Oxidase in Aqueous Solution

**DOI:** 10.1038/s41598-017-03177-y

**Published:** 2017-06-07

**Authors:** Qingju Liu, Li Chen, Zhikun Zhang, Bibai Du, Yating Xiao, Kunhao Yang, Lingling Gong, Li Wu, Xiangjun Li, Yujian He

**Affiliations:** 1 School of Chemistry and Chemical Engineering, University of Chinese Academy of Sciences, Huaibei Village, Huaibei Town, Huairou District, Beijing, 101408 China; 2 State Key Laboratory of Natural and Biomimetic Drugs, School of Pharmaceutical Sciences, Peking University, 38 Xueyuan Road, Haidian District, Beijing, 100191 China

## Abstract

D-amino acid oxidases (DAAO) are stereospecific enzymes which are generally almost inactive towards L-enantiomer in neutral solution when L-, D-amino acids are supplied as substrates. In this paper, the D-amino acid oxidase can catalytic oxidize L-amino acids by modulating pH of aqueous solution. With L-Pro as substrate, the catalytic rate (*k*_cat_) and the affinity (*K*_m_) of DAAO were 6.71 s^−1^ and 33 mM at pH 8.0, respectively, suggesting that optimal pH condition enhanced the activity of DAAO towards L-Pro. Similar results were obtained when L-Ala (pH 9.8), L-Arg (pH 6.5), L-Phe (pH 9.0), L-Thr (pH 9.4), and L-Val (pH 8.5) were catalyzed by DAAO at various pH values. The racemization of the L-amino acids was not found by capillary electrophoresis analysis during oxidation, and quantification analysis of L-amino acids before and after catalytic reaction was performed, which confirmed that the modulation of enantioselectivity of DAAO resulted from the oxidation of L-amino acids rather than D-amino acids by changing pH. A mechanistic model was proposed to explain enhanced activity of DAAO towards L-amino acids under optimal pH condition.

## Introduction

D-amino acid oxidase (DAAO) is the prototype of the FAD-dependent flavoprotein, which catalyzes oxidative deamination of a majority of D-amino acids rather than L-amino acids, producing the corresponding α-keto acid and ammonia^1–3^. DAAO is a ubiquitous enzyme which presents in numerous eukaryotic organisms^4^ and plays a crucial role in acting as a detoxifying agent of accumulated D-amino acids in the kidney and liver, maintaining the necessary levels of D-serine in different brain tissues^5^, and producing of 7-Aminocephalosporanic acid from the natural antibiotic cephalosporin C in a two-enzyme process^6^. Once enantioselectivity of DAAO is disturbed, the metabolic systems might be disordered, which might be found as precipitating factors of chiral disease.

DAAO has been extensively investigated over the past 80 years, but most issues focused on determining the structure of its binding to targeting protein^7, 8^, increasing its catalytic activity^9^ as well as exploring the probably correlation between the DAAO and the human diseases^10^. Recent advancements in utilization of DAAO include as biocatalysts for resolving racemic amino acid mixtures, as a tool for biosensing, a new mechanism of herbicide resistance^11–13^ and enzymatic synthesis of chiral amine^14, 15^. However, altering enantioselectivity of DAAO has been regarded as one of the most remarkable topics in enzymatic reactions, since the stereochemical outcome of a given enzyme-substrate reaction is predetermined in specific target environment. It is demonstrated that the alteration of enzyme enantioselectivity can be achieved by directed evolution^16^, site-directed mutagenesis^17–19^ and reaction temperature^20–22^. They require either a time-consuming enzymatic preparation or bring about undesirable deviation from the initial substrate. Additionally, the variation of the nature of the solvent greatly affected the enzyme enantioselectivity. For example, Margolin^23^ discovered that a facile method for enzymatic preparation of diverse peptides containing D-amino acids by radically altering enantioselectivity of enzymes in organic solvents. Cerovsky *et al*.^24^ reported that D-amino acid amides was incorporated into model peptides catalyzed by serine proteinase in acetonitrile with low water content. Tawaki *et al*.^25^ found a complete reversal of enantioselectivity of the transesterification catalyzed by *Aspergillus oryzae* protease with 18 anhydrous solvents. These studies have clearly shown that the changes of enzyme enantioselectivity can be achieved by using organic solvents, whereas little attention has been focused on the exploitation of aqueous solution, which is more important for the living systems.

Water is convenient to maintain the conformation of the enzyme catalytic center via the formation of hydrogen bonds, hydrophobic bond and Van der Waals force^26^. In aqueous solution, pH has a predominant influence on the enzymatic catalysis and can be easily controlled and manipulated. What’s more, appropriate pH conditions are essential for the ionic groups around the enzyme active center to achieve the best ionic state that enzyme catalytic reaction required. Up to date, much of researches have focused on the influence of pH on the activity and stability of enzyme, while few studies have looks at the issue of pH-dependent enantioselectivity. Just one typical example is reduction reaction of 2-butanone catalyzed by horse liver alcohol dehydrogenase, which exhibits the changes of pH-induced enantioselectivity and determine how the discrimination of substrates was affected^27^. Furthermore, Francesco^28^ developed a theoretical model based on differential catalytic commitments and to explain effect of pH on the reaction catalyzed by horse liver alcohol dehydrogenase and secondary alcohol dehydrogenase from *thermoanerobacter ethanolicus*.

Enzyme plays an important role in metabolism. In view of strict enantioselectivity and broad substrate specificity of DAAO to amino acids, a pH screening along with both enantiomers of amino acids was performed to study the influence of pH on enantioselectivity of the enzyme. Here, aqueous solution was used in enzymatic reaction. The results demonstrated that DAAO activities towards some neutral and basic L-amino acid were obviously improved by increasing or decreasing pH values when compared with their catalytic activities in neutral aqueous solution. Also, the DAAO activity towards acidic L-Asp was initiated and became quite similar towards both enantiomers of the acidic amino acids. Furthermore, catalytic oxidation of L-amino acids by DAAO was further confirmed by excluding the configuration inversion of L-amino acids and quantifying consumption of L-amino acids in the reaction process. Molecular modeling and optimization suggested that the enhanced catalytic activity of DAAO towards L-Ala at pH 9.8 may be due to the easy transpositions of amino group and methyl group of L-Ala binding to DAAO when positively charged amino group was changed into electrically neutral amino group under alkaline conditions. Such a study is necessary to understand the structure of active center, especially the state of charge, contributing alteration of stereospecificity of DAAO, which may provide a positive guidance for asymmetric catalytic oxidation of chiral amino acids by DAAO in aqueous solution.

## Results

### Effect of pH on activity of DAAO with neutral and basic L-amino acids as substrate

It’s acknowledged that DAAO was highly enantioselective which showed a strong preference for the D-amino acids and possessed a negligible catalytic efficiency on L-amino acids under physiological conditions. DAAO could dehydrogenate D-amino acids yielding hydrogen peroxide, which could interact with DHBS and 4-AAP yielding purple red semiquinone^29^. According to this detection method, the catalytic activities of DAAO with various L-amino acids as substrate were studied in a pH range from 6.0 to 10.5, as shown in Supplementary Figures S1–S17, and the ratios of UV absorbance of L-amino acids and D-amino acids were derived for easy observation, as shown in Table 1.

**Table 1:** The comparison of activity of DAAO with L- and D-amino acid as substrate.

Substrate	Δ^*^									
	pH 6.0	pH 6.5	pH 7.0	pH 7.5	pH 8.0	pH 8.5	pH 9.0	pH 9.4	pH 9.8	pH 10.5
Pro	—	0.8	2.7	6.8	17.2	4.6	3.7	3.1	2.8	—
Ala	—	—	—	—	—	—	—	—	19.4	—
Arg	—	11.8	—	—	—	—	—	—	—	—
Phe	—	—	—	—	—	—	1.1	—	—	—
Val	—	—	—	—	—	3.0	—	—	—	—
Met	—	—	—	—	—	—	—	—	—	—
Ser	—	—	—	—	—	—	—	—	—	—
Leu	—	—	—	—	—	—	—	—	0.6	—
Thr	—	—	—	—	—	—	—	7.6	—	—
Trp	—	—	—	—	—	—	—	—	—	—
His	—	—	—	—	—	—	—	—	-	—
Lys	—	—	—	—	—	—	—	—	—	—
Cys	—	—	—	—	—	—	—	—	-	—
Asn	—	—	—	—	—	0.6	—	—	—	—
Gln	—	—	—	—	50	—	—	—	—	—
Asp	—	—	—	—	77.2	—	—	—	—	—
Glu	—	—	—	—	—	—	—	—	—	—

Δ^*^ = (OD_512_(P_L-AA_)- OD_512_(Control))/(OD_512_(P_D-AA_) − OD_512_(Control)) × 100, where OD_512_(P_L-AA_) and OD_512_(P_D-AA_) are the absorbance of the product at 512 nm with L-amino acid and D-amino acid as substrate, respectively; —, no data.

It was found that varying degrees of oxidation deamination of L-Pro occurred with the change of pH values. DAAO displayed a slight catalytic activity towards L-enantiomers, and the ratio of UV absorbance of L-Pro to D-Pro at 512 nm was less than 2.7 when pH of the solution ranging from 6.0 to 7.0. Unexpectedly, with increasing pH value to 7.5, the ratio of relative activity raised to 6.8, and it reached to a pleateu of activity when pH value was 8.0. Then, the catalytic activity of DAAO towards L-Pro instead decreased when increasing pH value from 8.0 to 10.5. Obviously, catalytic activity of DAAO towards L-Pro was most evident at pH 8.0 and the ratio of UV absorbance of L-Pro to D-Pro was up to 17.2 (Table 1). The determination of DAAO activities at various pH values by coupling method was using peroxidase, whose activity could also be influenced by pH. Therefore, 2,4-dinotrophenylhydrazine was used to further confirm the activities^30^, and the results were shown in Supplementary Figures S18–S34 and Table S2. The catalytic activities DAAO towards the amino acids at various pHs using 2,4-dinitrophenylhydrazine agreed with their corresponding results by a coupling method using horseradish peroxidase, indicating that horseradish peroxidase was undisturbed by pH within the test range.

Next, the kinetic parameters (*k*_cat_ and *K*_m_) of DAAO with both enantiomers of Pro as substrate were determined in a range from pH 7.5 to pH 8.5, as shown in Table 2, where *k*_cat_ and *K*_m_ reflected the ability to catalyze substrate and affinity of enzyme, respectively. When D-Pro was used as substrate, *k*_cat_ of DAAO at pH 8.0 was 107.30 s^−1^, increased 2.2-fold and 2.47-fold in catalytic ability compared with pH 7.5 and pH 8.5, respectively. The affinity of DAAO towards D-pro slightly increased at pH 8.0 based on *K*_m_ values. However, there was a significant increase (9.59-fold for pH 7.5 and 16.78-fold for pH 8.5) in catalytic activity of DAAO towards L-Pro at pH 8.0. Also, there was a tremendous increase in affinity of DAAO to substrate at pH 8.0, as evidenced by *K*_m_ values changing from 252 mM (pH 7.5) and 1800 mM (pH 8.5) to 20.00 mM (pH 8.0). Clearly, the increase in catalytic rate of DAAO to L-Pro was greatly different from that of DAAO to D-Pro when pH was changed into 8.0, thereby leading to only 15.99-fold drop in the catalytic activity according to *k*_cat,D-Pro_/*k*_cat,L-Pro_, while it occurred 71.42-fold and 108.25-fold drop at pH 7.5 and pH 8.5, respectively. Similarly, the relative catalytic ability (*k*_cat,D-Ala_/*k*_cat,L-Ala_) of DAAO to Ala tremendously decreased from 1680.00 at pH 7.0 to 14.18 at pH 9.8 (Table 3). However, it was reported that the initial rate of enzymatic deamination for D-amino acids was found to exceed that for L-amino acids by 3000 to 4000-fold under physiological condition^31^. Our results indicated that the changes of pH significantly enhanced the catalytic rate of DAAO towards L-Pro and L-Ala. Moreover, the observed relaxation of enantioselectivity of DAAO induced by pH was due to a much higher reactivity of the L-Pro rather than a much lower reactivity of its D-counterpart.

**Table 2:** Comparison of kinetic parameters of DAAO to D-Pro and L-Pro under different pH values.

pH	D-Pro		L-Pro		*k*_cat_(D-Pro) /*k*_cat_(L-Pro)
	*k*_cat_(s^−1^)	*K*_m_(mM)	*k*_cat_(s^−1^)	*K*_m_(mM)	
7.5	50.20 ± 1.20	0.38 ± 0.02	0.70 ± 0.06	252.00 ± 10.00	71.42
8.0	107.30 ± 9.10	0.33 ± 0.01	6.71 ± 0.51	20.00 ± 1.73	15.99
8.5	43.30 ± 3.40	0.85 ± 0.06	0.40 ± 0.03	1800.00 ± 90.50	108.25

**Table 3:** Comparison of kinetic parameters of DAAO to D-Ala and L-Ala under different pH values.

pH	D-Ala		L-Ala		*k*_cat_(D-Ala) /*k*_cat_(L-Ala)
	*k*_cat_(s^−1^)	*K*_m_(mM)	*k*_cat_(s^−1^)	*K*_m_(mM)	
7.0	151.20 ± 1.20	30.50 ± 1.50	0.09 ± 0.01	362.00 ± 10.00	1680.00
9.8	133.30 ± 3.40	38.20 ± 1.20	9.40 ± 0.39	43.11 ± 3.61	14.18

To check the phenomenon was common occurred for all amino acids, all L-amino acids except for L-Tyr were applied to check DAAO activity due to L-Tyr’s low solubility. Enzyme samples were prepared by dissolving the purified DAAO in different buffer solutions containing various concentrations of L-amino acids and activity was evaluated under standard assay conditions. Similar phenomena were observed under different pH conditions and the results were shown in Table 1 and Supplementary Tables S1 and S2. Obviously, pH variation strikingly accelerated the reaction of DAAO with L-Arg, L-Phe, L-Thr and L-Val, in addition to L-Pro and L-Ala, and even the *k*_cat_ is up to 9.4 s^−1^ for L-Ala under optimum pH conditions (Supplementary Table S1). In contrast, the influence of pH on DAAO activity was modest in presence of L-Leu, L-Asn, and L-Gln. However, in case of L-Ile, L-Ser, L-Lys, L-His, L-Cys, L-Met and L-Trp, the activities were negligible and thus the ratio in absorbance of L-amino acid and D-amino acids was not obtained by the same measure method (Table 1 and Tables S2). These results indicated that pH variation perturbed the regular enantioselectivity of DAAO towards some amino acids.

### Effect of pH on activity of DAAO with acidic L-amino acid as substrate

As we know, DAAO possesses very poor or no activity towards D-Asp and D-Glu under normal physiological conditions since D-asparate oxidase oxidizes D-enantiomer of acidic amino acids in more specific fashion. However, it’s wondered whether pH variation would increase DAAO activity when acidic L-amino acids were used as substrates.

The enantiomers for Glu and Asp were parallelly assayed under the various pH conditions and the results were shown in Table 4. As inactive as DAAO went, the catalytic efficiency of DAAO to both D-Glu and L-Glu was unnoticeable regardless of what kind of pH condition. Nevertheless, DAAO exhibited activity towards not only L-Asp but also D-Asp when pH was changed into 8.0. Moreover, DAAO possessed a similar catalytic activity (*k*_cat,L-Asp_ = 0.89) for L-Asp compared to with D-Asp (*k*_cat,D-Asp_ = 0.95), as well as similar affinity (*K*_m,D-Asp_ = 43 and *K*_m,L-Asp_ = 45). These results confirmed that pH variation could initiate activity of DAAO towards certain acidic L-amino acid.

**Table 4:** Comparison of kinetic parameters of DAAO with Asp and Glu as substrate at pH 8.0. —, no data.

Substrate	*k*_cat_(s^−1^)	*K*_m_ (mM)
D-Asp	0.95 ± 0.08	43 ± 4
L-Asp	0.89 ± 0.07	45 ± 5
D-Glu	—	—
L-Glu	—	—

### Confirmation for catalytic oxidation of L-amino acids in the catalytic process

**Figure 1:**
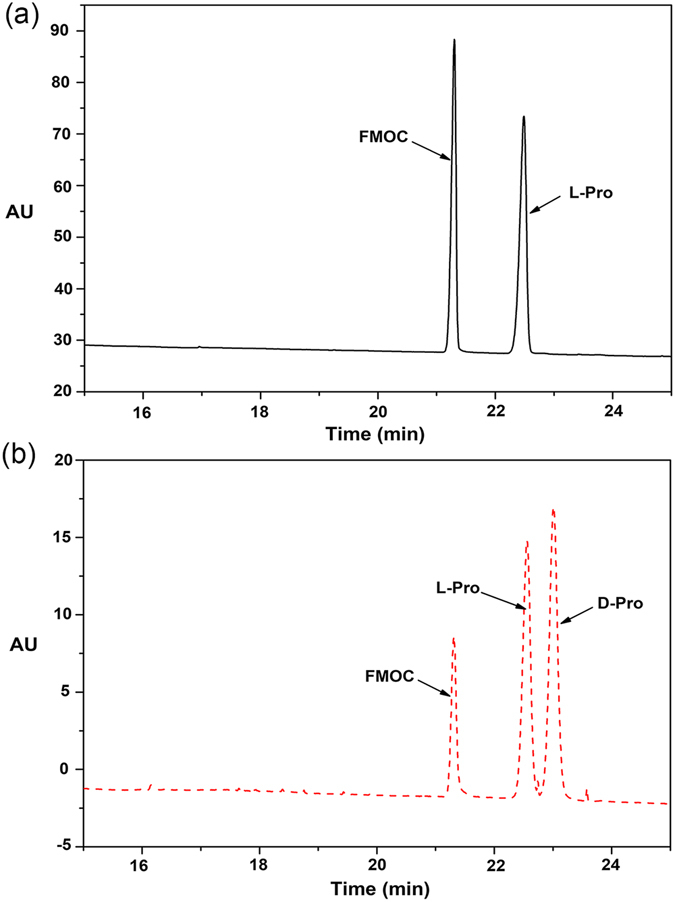
CE analysis of solution of derivatized L-Pro (**a**) and the mixture solution of L-Pro and D-Pro after derivatization (**b**) after 30 min incubation at pH 8.0 at 37 °C.

Since racemization of amino acids is easy to occur in alkaline solution or when heated^32^, it’s obviously impossible to distinguish whether DAAO catalyzed the oxidative deamination of L-amino acids under special conditions or DAAO catalyzed the oxidative deamination of D-amino acids arising from the racemization of L-amino acids during the experiments. If the racemization occurred during the reaction, how much contamination of D-amino acid over L-amino acid would result in visualizing the oxidative deamination of L-amino acid by DAAO. Thus, to exclude racemization of L-amino acid, we chose some L-amino acids able to be catalyzed by DAAO (Fig. 1 and Supplementary Figure S35).

After 30 minutes incubation at 37 °C, the solution of L-Pro (pH 8.0) was derived by the FMOC and then analyzed on CE, which was capable of differentiating the derivatives of D- and L-amino acids with high resolution and efficiency. The derivative of D-Pro was not detected in the solution of derivatized L-Pro after 30 min incubation in the detection limit range (10^−9^ mol/L), according to the comparation of L-Pro solution and the mixture solution of L-Pro and D-Pro after derivatization (Fig. 1), which indicated that the L-Pro was not transformed to its mirror-image configuration at pH 8.0 and DAAO indeed catalyzed the oxidation of L-Pro during the reaction time. Similarly, solutions of the other L-amino acids solutions such as Ala, Phe, Gln, Arg, Leu, Thr, Asp, Asn and Val were also analyzed under their optimal pH conditions, and it was found that D-enantiomers were not detected within reaction time (Supplementary Figure S35).

Although the initial activity of DAAO to L-amino acids and D-amino acids was determined by UV (Supplementary Figure S1–S34) and HPLC analysis (Supplementary Figure S36), the quantitive analysis of catalytic oxidation of L-amino acids by DAAO was further performed. The contents of L-amino acids before (S_before_) and after reaction (S_after_) with DAAO were examined by CE analysis and the result were listed in Table 5. DAAO and L-Pro were incubated in the phosphate buffer (20 mM) at pH 7.0 and pH 8.0, respectively. As a control, no change in content of L-amino acids was observed at pH 7.0. However, when DAAO and L-Pro were incubated at pH 8.0, the content of L-Pro after reaction accounts for 17.16% that of before reaction based on the peak area of derived L-Pro. Similarly, the amounts of the other amino acids (L-Ala, L-Arg, L-Phe, L-Gln, L-Leu, L-Thr, L-Asp, L-Asn and L-Val) obviously decreased after incubation with DAAO at 37 °C for 30 min under optimum pH conditions, which agreed with changes of UV absorbance in oxidizing the L-amino acids. Moreover, the conversion rates of the L-amino acids were different from each other, due to different interactions between enzyme and various the L-amino acids, such as substrate binding, hydrophobic binding and reactive potential of active sites^33^. The reduction in content of L-amino acids with catalytic oxidation of DAAO was also an evidence for the increasing of DAAO activity to L-amino acids under optimal pH conditions.

**Table 5:** Quantification of L-amino acids before and after reaction of DAAO by CE analysis.

pH	L-amino acid	S_before_	S_after_	(S_before_ − S_after_)/S_before_(%)
8.0	L-Pro	38880.83	33545.33	17.16
9.8	L-Ala	18084.72	15603.50	13.72
6.5	L-Arg	32483.21	27603.50	15.02
9.0	L-Phe	36132.83	29223.80	9.12
8.0	L-Gln	17798.74	17711.53	0.49
9.8	L-Leu	22968.91	22819.61	0.65
9.4	L-Thr	11935.17	11036.45	7.53
8.0	L-Asp	12307.99	12251.37	0.46
8.5	L-Asn	9707.83	9652.49	0.57
8.5	L-Val	12108.88	11728.66	3.14

## Discussion

Change of pH might induce conformational variations of DAAO as well as other effects such as protonation state of amino acid residues at the active site and interaction between active site of DAAO and substrate. Thus, a structural understanding is helpful to explain relaxation of enantioselectivity of DAAO. We first investigated the conformation of DAAO solution in various pH conditions by CD analysis, since CD spectrum analysis was utilized to test whether pH induced a disruption of the overall protein structure or just indicative of local unfolding. The stability studies of DAAO were characterized by the CD signal around 215 nm (taken as a reporter of the change in secondary structure)^34^ and depicted in Supplementary Figure S37. DAAO is a type of βαβ protein under physiological condition, as evidenced by a clear negative peak around 215 nm^35^. Moreover, CD signal at 215 nm became slightly stronger with pH of DAAO solution increasing from 6.5 to 10.0, but no shift in wavelength was observed (Supplementary Figure S37b), indicating that there occurred a minor alteration from α to β for DAAO conformation in the pH range. However, CD signal at 215 nm shifted to 222 nm when pH of the DAAO solution increased to 10.5, suggesting a relative larger change for secondary structure formed, as shown in Supplementary Figure S37a The interesting phenomena was consistent with experimental data that much activity to L-Ala at pH 9.8 but not completely at pH 10.5 (Supplementary Figure S2).

To further explain why DAAO’s enantioselectivity is pH-dependent as shown in Tables 1,2 and 3, the plausible structures of DAAO in complex with D- and L-enantiomers of an amino acid were deduced by molecular optimization. The DAAO utilized in the experiments is a domain from porcine kidney with 347 amino acid residues, which is designated pkDAAO. The active site of pkDAAO is made up of five amino acids: Tyr224, Tyr228, Arg283, Gly313 and Gln53^36^, which would be vital for the binding and orientation of the substrate.

**Figure 2:**
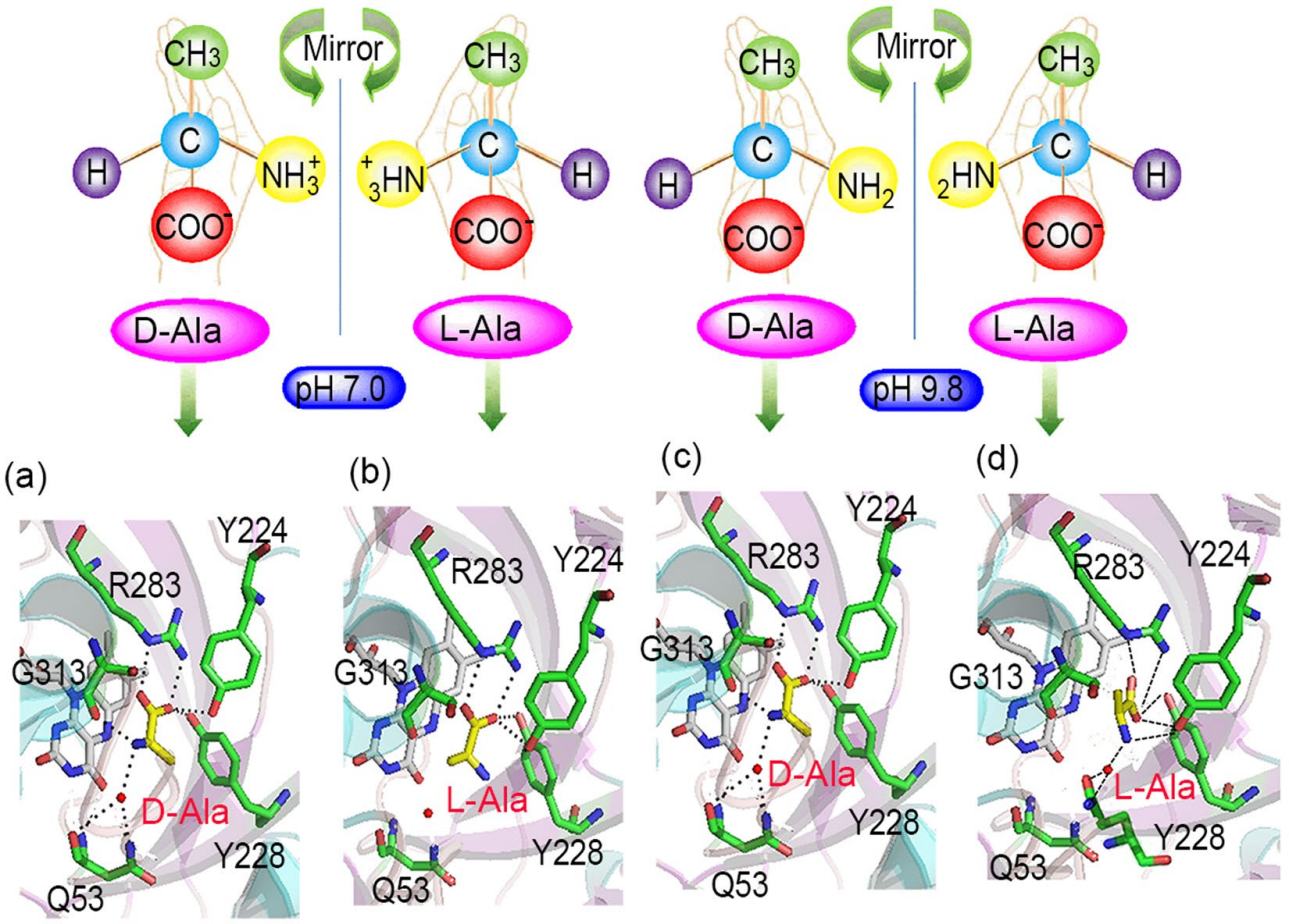
Graphical presentation of the active site cavity of DAAO in complex with both enantiomers of Ala at different pH. The active site residues, the L-Ala and D-Ala molecule were shown in stick representation colored in green (carbon atoms), blue (nitrogen atoms) and red (oxygen atoms). The molecular modeling displayed DAAO in complex with D-Ala at pH 7.0 (**a**), DAAO in complex with L-Ala at pH 7.0 (**b**), DAAO in complex with D-Ala at pH 9.8 (**c**), DAAO in complex with L-Ala at pH 9.8 (**d**), respectively.

The model of binding of D-Ala to pkDAAO under physiological conditions is presented in Fig. 2a and Supplementary Figure S38. Also make special note that the amino group of Ala is positively charged in near-neutral pH solution, and the interaction between DAAO and D-enantiomer is described as follows^36, 37^: (a) The α-COO^−^ of the substrate makes a salt bridge with the cationic guanidine group of Arg283 and one of its oxygen atoms interacts with the hydroxyl group of the two tyrosine residues (Tyr224 and Tyr228); (b) The NH_3_^+^ of substrate is hydrogen bond with the backbone carbonyl of Gly313, with a tendency to ionic bonding, and also bound to Gln53 by a water molecule in between, whereas the water molecule has well-defined electron density in all eight subunits present in the asymmetric unit, which helps H_2_O to attack α-amino group during deaminization. Rather, D-Ala is tightly bound to the active site of the enzyme by noncovalent interaction, forming a quite stable transient intermediate. However, L-Ala, as mirror image of D-Ala, has both NH_3_^+^ and CH_3_ but in the reversed position, which are placed into the binding site cavity in terms of same steric bulk^38, 39^. The L-enantiomer of Ala binds to the enzyme in a fashion shown in Fig. 2b. The α-COO^−^ substituents at the chiral carbon bind to the Arg283, Tyr224 and Tyr228 the same way as those of its D-counterpart, whereas the amino group faces away from the carbonyl moiety of Gly313 and Gln53, thereby making the nucleophilic attack impossible, which would explain why the pkDAAO is high stereospecific to D-Amino acids under physiological conditions.

When pH of the near-neutral solution rised to 9.8, the cationic amino group could be deprotonated to become neutral amino group-NH_2_, forming the hydrophobic environment surrounding. The function groups -NH_2_ and CH_3_ of L-Ala were isosteres, which shared similar electronegativities and biological activities. The steric difficulties encountered by the D- or L-enantiomer will not be exacerbated, then either of Ala would be enough to fit into the pocket of enzyme active center. Thus, the productive binding between α-COO^−^ substituents and the amino acids (Arg283, Tyr224 and Tyr228) of enzyme active center will be unaffected for both D- and L-enantiomer in spite of an increase of pH, whereas the hydrogen binding formed by amino group’s nucleophilic attack carbonyl of Gly313 weakened due to change of α-amino group of Ala from NH_3_^+^ to NH_2_. Interestingly, there remains one hydrogen binding comes into being between H_2_O and α-amino group for the L-enantiomer, in addition to hydrogen binding between the amino group and Gln53 through coupling with one H_2_O for the D-Ala. It is reported that the reaction of ALa catalyzed by DAAO first dehydrogenates the amino acid to the corresponding imino acid, coupled with the reduction of FAD, then hydrolyzes to the a-keto acid and ammonia^1^. Thus, the attack of H_2_O to α-amino group play a key role in catalyzed oxidization of amino acid. The weakened interaction between substrate and active site of enzymic cavity at pH 9.8 in comparasion to the near-neutral condition, leads to easy rotation of Ala and thus increases attacking chances of H_2_O to α-amino group of L-Ala (Fig. 2b and d). In addtion, the efficient hydride transfer of substrate to FAD is also crucial for the dehydrogenation of amino acid. At high pH (≥8), the substrate amino acid binds in the “ α-NH_2_ form“ that is suitable for immediate hydride transfer. After pH ≤ 7 (α-NH_3_^+^ form), the H^+^ liberated during hydride transfer is proposed to be relayed to bulk solvent^40^. Indeed, the catalytic activity (*k*_cat_) of the DAAO to L-Ala at pH 9.8 increases from 0.09 s^−1^ to 9.40 s^−1^, and the binding affinity of the DAAO for L-Ala is relatively altered (Table 3). Clearly, the relaxation of stereospecificity of DAAO occurred from D- to L-enantiomer when the pH increases from 7.0 to 9.8. This has been verified experimentally: Upon transition from pH 7.0 to pH 9.8, (*k*_cat_)_L-Ala_ increases more than 104.4-fold, whereas (*k*_cat_)_D-Ala_ decreases only 1.1-fold, thereby leading to the overall 114.8-fold drop in enantioselectivity (Table 3).

In the case of Pro, the group –CH_2_- and –NH- attached to chiral carbon atom are also isosteres like Ala, and further the five membered heterocyclic conformation containing chiral carbon at the side chain of Pro formed a compact and unencumbered structure so that both enantiomers of Pro would fit well with cavity of active center of DAAO, and thus the reason for relaxation of enantioselectivity of DAAO to Pro may be the same as the explanation for modeling of DAAO binding to Ala.

Previous studies have shown that the reaction catalyzed by DAAO proceeds without direct involvement of amino acid functional groups^13^. Most residues present at the active site are involved in substrate binding and precise orientation of the interacting orbitals^13^, the remarkable substrate specificity change mainly ascribes to new favorable electrostatic interactions between the guanidium group of the L-Arg and the negative charge of the active center. The ε-NH_2_ and –NH_2_- of Arg is positively charged in neutral, basic and even most basic environments. The electrostatic interaction between ε-NH_2_ and –NH_2_- and active center of enzyme in presence of pH 6.5 is stronger than in pH 7.5, thus electrostatic interactions may facilitate the orientation of L-Arg by DAAO, resulting in activation to L-Arg. Conformational diversity of the active site and substrate is likely to contribute to acceptance of non-native compounds in many cases^33^. Due to the the flexibility of active site, the electrostatic interactions between the COO^−^ of side chain and enzyme help the recognization of L-Asp by DAAO. In contrast, the longer side chain of L-Glu displays increased steric exclusion from an active site.

In view of different pk_a1_ and pk_a2_ corresponding to different amino acids (Pro, Ala, Arg, Thr, Val, Tyr and Phe), the amino acids residues in the active center and the substrates are in different ionic and charge states. Therefore, the L-configuration of different amino acids could be recognized by DAAO under different pH conditions, exhibiting optimal activity at different pH values. However, for most amino acids, despite pH is able to change the protonated state of substrate, there is striking disparity in the function group of the side chain, thereby showing no activation by DAAO. One possible reason is that the reversed groups of amino acids experiences severe steric hindrances in fitting into the binding pocket, which agreed with the fact that the active sites of hydrophobic resides of pkDAAO formed a cavity roughly corresponding to the volume occuped by an amino acid with a four-carbon-atom side chain^1^.

## Conclusions

In summary, the study demonstrated that a biochemically useful process, an enzymatic enantioselectivity of amino acids with DAAO, could be controlled by easily adjusting the pH of aqueous solution, although the adjustment of pH was not always in good agreement with our predictive physiological conditions. The model that catalytic oxidation of DAAO to all the amino acids was developed and confirmed experimentally, and this phenomenon was further mechanistically explained by molecular optimization. In addition, it should be noted that pH control of enantioselectivity is not limited to DAAO. Actually, our preliminary study with aminoacyl-tRNA synthetase is to predict that its enantioselectivity in transesterification of amino acids also depends on the environmental factors, even though the catalyzed enantioselective reaction of DAAO towards amino acids is opposite to that of the aminoacyl transferase. On a practical note, these data (A pH screening along with a conventional biocatalyst) exhibit the usefulness and generality of incorporating the pH optimization step in the overall strategy of the biocatalytic asymmetric resolutions.

## Methods

### Materials

L- and D-amino acids, 9-fluorenylmethyl chloroformate (FMOC), sodium 3, 5-dichloro-2-hydroxybenzenesulfonate (DHBS), 4-aminoantipyrine (4-AAP) and 2,4-dinitrophenylhydrazine were purchased from J&K Scientific. D-Amino acid oxidase from porcine kidney (EC 1.4.3.3) and peroxidase (POD) were obtained from Sigma-Aldrich. All other chemicals were obtained from Aladdin.

### Kinetics measurements at different pH values with L-amino acids as substrate

The activity of DAAO on L-amino acids was determined according to improved hydrogen peroxide determination via the coupled peroxidase assay^41^. The effect of pH on the enantioselectivity of DAAO was determined by incubating the reaction mixture at pH values ranging from 6.0 to 10.5. In order to meet the desired range in pH, different buffers were utilized: 20 mM sodium phosphate buffer solutions (pH 6.0–8.0); 20 mM sodium borax buffer (pH 8.5–10.5). All buffers were adjusted to the desired pH at the given reaction temperatures and detected by PB-pH meter (Sartorius, German). All experiments were carried out for 30 min at 37 °C in 1 mL reaction mixtures containing 228 U/L DAAO, 300 U/L POD, 0.275 nM 4-AAP, 5.5 mM DHBS, 0.1 mM FAD and various concentrations of L- or D-amino acids at different pH level. Amino acids were consumed from control mixtures. Afterward, 200 μL 6% (w/v) ice-cold trichloroacetic acid was added to the incubated mixtures and centrifuged at 12000 rpm for 10 min. Then the supernatant was determined by UV spectrophotometer (Shimadzu UV-2550) and the absorbance of the supernatant was measured at 512 nm against a blank sample consisting of the same mixture without amino acids. The activity of DAAO in the mixtures was quantified against the standard DAAO curves (from 3–54 units/L, r > 0.97).

### Quantification of L-amino acids before and after reaction

Capillary electrophoresis (CE) method conditions were carried out on Beckmann MDQ-3063847. 20 mM borax buffer solution (pH 9.2) containing 30 mM β-CD, 25 mM SDS and 17% IPA was used as the electrolyte. The effective length and total length of the capillaries were 47 and 60 cm, respectively. Detection at 210 nm was performed. Sample solutions were introduced at the cathodic end at a pressure of 20 KV.

Prior to sample introduction, the capillary was flushed with 100 mM NaOH, water and running electrolyte for 2 min, respectively. 1 mL reaction mixtures containing 228 U/L DAAO and 25 mM L-Pro were incubated for 30 min at 37 °C. After derivatization with FMOC, an aliquot (10 μL) of the sample was diluted and injected into the CE apparatus for the quantification of L-amino acids before and after reaction. Derivatization of amino acids with FMOC was as described earlier^42^. Briefly, 200 μL of 10 mM FMOC was added to 200 μL of amino acids in 20 mM borate buffer (pH 9.0). This mixture was kept for 2 min, and then extracted with 0.5 mL pentane to remove excess of reagent. After dilution ten times with water, the sample was ready for introduction.

### Detection of racemization of amino acid

After incubation at 37 °C for 30 min, 1 mL L-Pro solution (25 mM) prepared with sodium borax buffer (pH 8.0) was divided into two parts. One of them was for the CE analysis after derivatization with FMOC. D-Pro of the same concentration was added to the other part and was for the detection of racemization of amino acids after derivatization with FMOC. Derivatization of the amino acids was carried out according to the above methods. L-Ala (pH 9.8), L-Arg (pH 6.5) and L-Phe (pH 9.0) were tested under the same condition.

### Circular dichroism (CD) spectroscopy

CD spectroscopic studies were performed using a Jasco-815 spectrophotometer (Jasco, Japan) equipped with a Peltier system for controlling the temperature. The sample was pre-equilibrated at 25 °C for 15 min and the scan speed was fixed for adaptative sampling (error F 0.01) with a response time of 1 s and 1 nm bandwidth. The structures of DAAO were monitored by using 1.0 cm path length cuvette. The concentration DAAO was 0.4 mg/mL. Each sample spectrum was obtained by appropriate blank media without DAAO from the experimental enzyme spectrum.

### Structure modeling of DAAO

Swissmodel (http://swissmodel.expasy.org) was used to predict structure by indentifying pkDAAO structural homologues. Homology modeling was performed with use of the crystal structure of the DAAO (PDB code 1COP) as a template. The ligand geometries of the 3D structure were optimized by using MMFF forcefield and MMFF charges for the atoms, till a gradient of 0.001 kcal/mol/Å was reached, maintaining the template structure rigid during minimization. The modeling of the enzyme was performed in the presence and absence of two buffers (pH 7.0 and pH 9.8). Molecular graphics were constructed by PyMOL.

## Electronic supplementary material

Supporting Information

## References

[1] Pilone MS (2000). D-Amino acid oxidase: new findings. Cell Mol Life Sci.

[2] Daniello A (1993). Biological role of D-amino acid oxidase and D-aspartate oxidase. Effects of D-amino acids. J Biol Chem.

[3] Tishkov VI, Khoronenkova SV (2005). D-Amino acid oxidase: structure, catalytic mechanism, and practical application. Biochemistry (Mosc).

[4] Friedman M (1999). Chemistry, nutrition and microbiology of D-amino acids. J Agric Food Chem.

[5] Smith SM, Uslaner JM, Hutson PH (2010). The Therapeutic Potential of D-Amino Acid Oxidase (DAAO) Inhibitors. Open Med Chem J.

[6] Pollegioni L, Sacchi S, Pilone MS, Mola G (2007). Physiological functions of D-amino acid oxidases: from yeast to humans. Cell Mol Life Sci.

[7] Chang SL (2014). The C-terminal region of G72 increases D-amino acid oxidase activity. Int J Mol Sci.

[8] Tran DH (2015). Identification of DNA-binding proteins that interact with the 5’-flanking region of the human d-amino acid oxidase gene by pull-down assay coupled with two-dimensional gel electrophoresis and mass spectrometry. J Pharmaceut Biomed.

[9] Cappelletti P (2015). Structure-function relationships in human d-amino acid oxidase variants corresponding to known SNPs. BBA-Proteins and proteom.

[10] Ma S, Li XY, Gong N, Wang YX (2015). Contributions of spinal d-amino acid oxidase to chronic morphine-induced hyperalgesia. J Pharmaceut Biomed.

[11] Tishkov VI, Khoronenkova SV (2005). D-amino acid oxidase: structure, catalytic mechanism, and practical application. Biochemistry (Moscow).

[12] Pollegioni L, Piubelli L, Sacchi S, Pilone MS, Molla G (2007). Physiological functions of D-amino acid oxidases: from yeast to humans. Cell Mol Life Sci.

[13] Pollegioni L, Molla G (2011). New biotech applications from evolved D-amino acid oxidases. Trends biotechnol.

[14] Yasukawa K, Nakano S, Asano Y (2014). Tailoring D-AminoAcid Oxidase from the Pig Kidney to R-Stereoselective Amine Oxidase and its Use in the Deracemization of α-Methylbenzylamine. Angew Chem Int Edit.

[15] Nakano S (2016). Origin of Stereoselectivity and Substrate/Ligand Recognition in an FAD-Dependent R‑Selective Amine Oxidase. J Phys Chem B.

[16] Zha, D. X., Wilensek, S., Hermes, M., Jaeger, K. E., & Reetz, M. T. Complete reversal of enantioselectivity of an enzyme-catalyzed reaction by directed evolution. *Chem Commun*, 2664–2665 (2001).

[17] Bartsch S, Kourist R, Bornscheuer UT (2008). Complete inversion of enantioselectivity towards acetylated tertiary alcohols by a double mutant of a Bacillus subtilis esterase. Angew Chem Int Edit.

[18] Bordes F (2009). Improvement of Yarrowia lipolytica lipase enantioselectivity by using mutagenesis targeted to the substrate binding site. Chembiochem.

[19] Lin H, Tang DF, Ahmed AAQ, Liu Y, Wu ZL (2012). Mutations at the putative active cavity of styrene monooxygenase: Enhanced activity and reversed enantioselectivity. J Biotechnol.

[20] Jin X, Liu BK, Ni Z, Wu Q, Lin XF (2011). A novel control of enzymatic enantioselectivity through the racemic temperature influenced by reaction media. Enzyme and Microb Tech.

[21] Massey V, Curti B, Ganther H (1966). A temperature-dependent conformational change in D-amino acid oxidase and its effect on catalysis. J Biol Chem.

[22] Lutz-Wahl S, Trost EM, Wagner B, Manns A, Fisher L (2006). Performance of D-amino acid oxidase in presence of ionic liquids. J Biotechnol.

[23] Margolin AL, Tai DF, Klibanov AM (1987). Incorporation of D-amino acids into peptides via enzymatic condensation in organic-solvents. J Am Chem Soc.

[24] Cerovsky V (1991). Serine proteinase-catalyzed incorporation of D-amino acids into model peptides in acetonitrile with low water-content. Biomed Biochimica Acta.

[25] Tawaki S, Klibanov AM (1992). Inversion of enzyme enantioselectivity mediated by the solvent. J Am Chem Soc.

[26] Fitzpatrick PA, Klibanov AM (1991). How can the solvent affect enzyme enantioselectivity. J Am Chem Soc.

[27] Adolph HW, Kiefer M, Zeppezauer M (1993). The influence of pH on the substrate-specificity and stereoselectivity of Alcohol-Dehydrogenase from Horse Liver. Enzymology and Molecular Biology of Carbonyl Metabolism.

[28] Secundo F, Phillips RS (1996). Effects of pH on enantiospecificity of alcohol dehydrogenases from *Thermoanaerobacter ethanolicus* and horse liver. Enzyme Microb Tech.

[29] Gabler M, Hensel M, Fischer L (2000). Detection and substrate selectivity of new microbial D-amino acid oxidases. Enzyme Microb Tech.

[30] Pollegioni L, Servi S (2012). Unnatural amino acids methods and protocols. Methods Mol Biol.

[31] Wellner D, Scannone H (1964). Oxidation of L-proline and L-3,4-dehydroproline by D-amino acid oxidase. Biochemistry.

[32] Miyamoto T (2015). Origin of d-amino acids detected in the acid hydrolysates of purified Escherichia coli – galactosidase. J Pharmaceut Biomed.

[33] Babtie A, Tokuriki N, Hollfelder F (2010). What makes an enzyme promiscuous?. Curr Opin Chem Biol.

[34] Miura R (2001). Porcine kidney D-amino acid oxidase: the three-dimensional structure and its catalytic mechanism based on the enzyme-substrate complex model. J Mol Catal B-Enzym.

[35] Caldinelli L (2004). Unfolding intermediate in the peroxisomal flavoprotein D-amino acid oxidase. J biol chem.

[36] Pollegioni L, Fukui K, Massey V (1994). Studies on the kinetic mechanism of pig-kidney D-amino acid oxidase by site-directed mutagenesis of Tyrosine-224 and Tyrosine-228. J Biol Chem.

[37] Mattevi A (1996). Crystal structure of D-amino acid oxidase: a case of active site mirror-image convergent evolution with flavocytochrome b2. P Natl Acad Sci USA.

[38] Mizutani H (1996). Three-dimensional structure of porcine kidney D-amino acid oxidase at 3.0 Å resolution. J Biochem.

[39] Setoyama C (1996). Crystallization of expressed porcine kidney D-amino acid oxidase and preliminary X-ray crystallographic chatacterization. J Biochem.

[40] Umhau S (2000). The X-ray structure of D-amino acid oxidase at very high resolution identifies the chemical mechanism of flavin-dependent substrate dehydrogenation. P Natl Acad Sci USA.

[41] Martinez-Martinez I, Navarro-Fernandez J, Garcia-Carmona F, Sanchez- Ferrer A (2008). Implication of a mutation in the flavin binding site on the specific activity and substrate specificity of glycine oxidase from *Bacillus subtilis* produced by directed evolution. J Biotechnol.

[42] Ziegler J, Abel S (2014). Analysis of amino acids by HPLC/electrospray negative ion tandem mass spectrometry using 9-fluorenylmethoxycarbonyl chloride (Fmoc-Cl) derivatization. Amino Acids.

