# Supplementary material for: PH-Dependent Enantioselectivity of D-amino Acid Oxidase in Aqueous Solution
Source: Sci Rep. 2017 Jun 7;7:2994. doi: 10.1038/s41598-017-03177-y (PMC5462808; doi:10.1038/s41598-017-03177-y)
Supplement: Supplementary file 1 — Supporting Information [file 41598_2017_3177_MOESM1_ESM.pdf]

PH-Dependent Enantioselectivity of D-amino Acid Oxidase in Aqueous Solution

Qingju Liu[a], Li Chen[a], Zhikun Zhang[a], Bibai Du[a], Yating Xiao[a], Kunhao Yang[a], Lingling Gong[a], Li Wu[a*], Xiangjun Li[a*], Yujian He[a,b*]

[a]School of Chemistry and Chemical Engineering , University of Chinese Academy of Sciences, Beijing, 101408, PR China.

[b]State Key Laboratory of Natural and Biomimetic Drugs, Peking University, Beijing, 100191, PR China

[*]Corresponding author:

E-mail: wuli@ucas.ac.cn; lixiangj@ucas.ac.cn; heyujian@ucas.ac.cn.

Tel/Fax: (010) 69672529

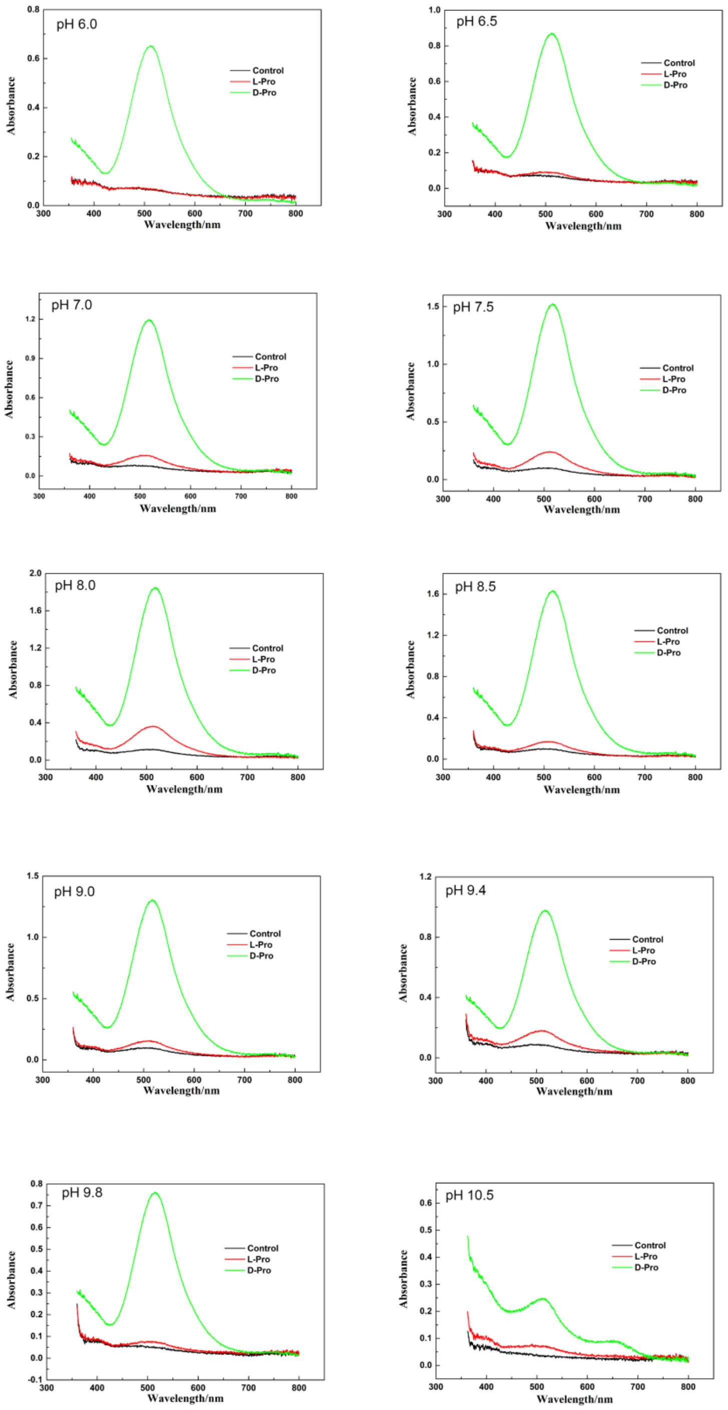

Figure S1 UV absorbance of UV-vis spectrum of the reaction product derived from DAAO and L-, D-Pro, the concentration of L-Pro and D-Pro was 25 mM.

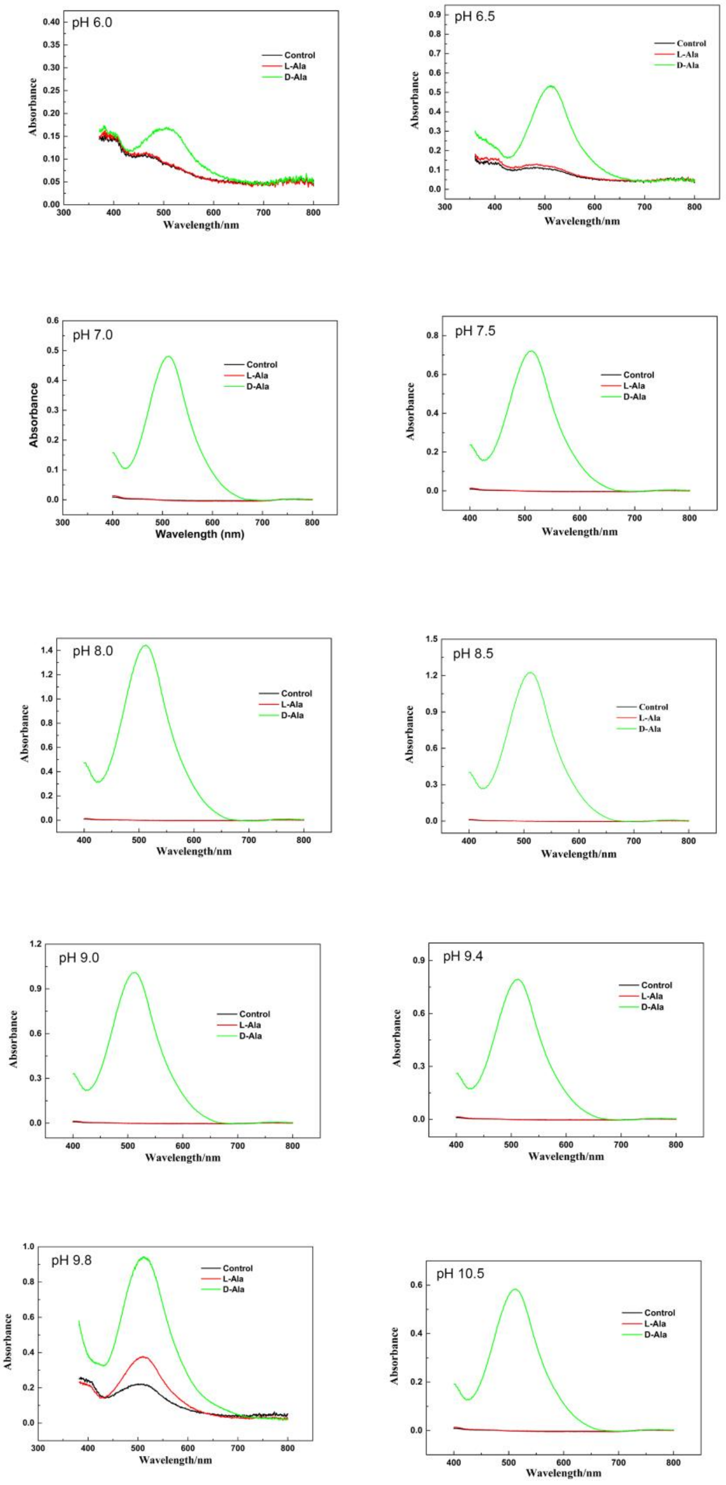

Figure S2 UV absorbance of UV-vis spectrum of the reaction product derived from DAAO and L-, D-Ala, the concentration of L-Ala and D-Ala was 25 mM.

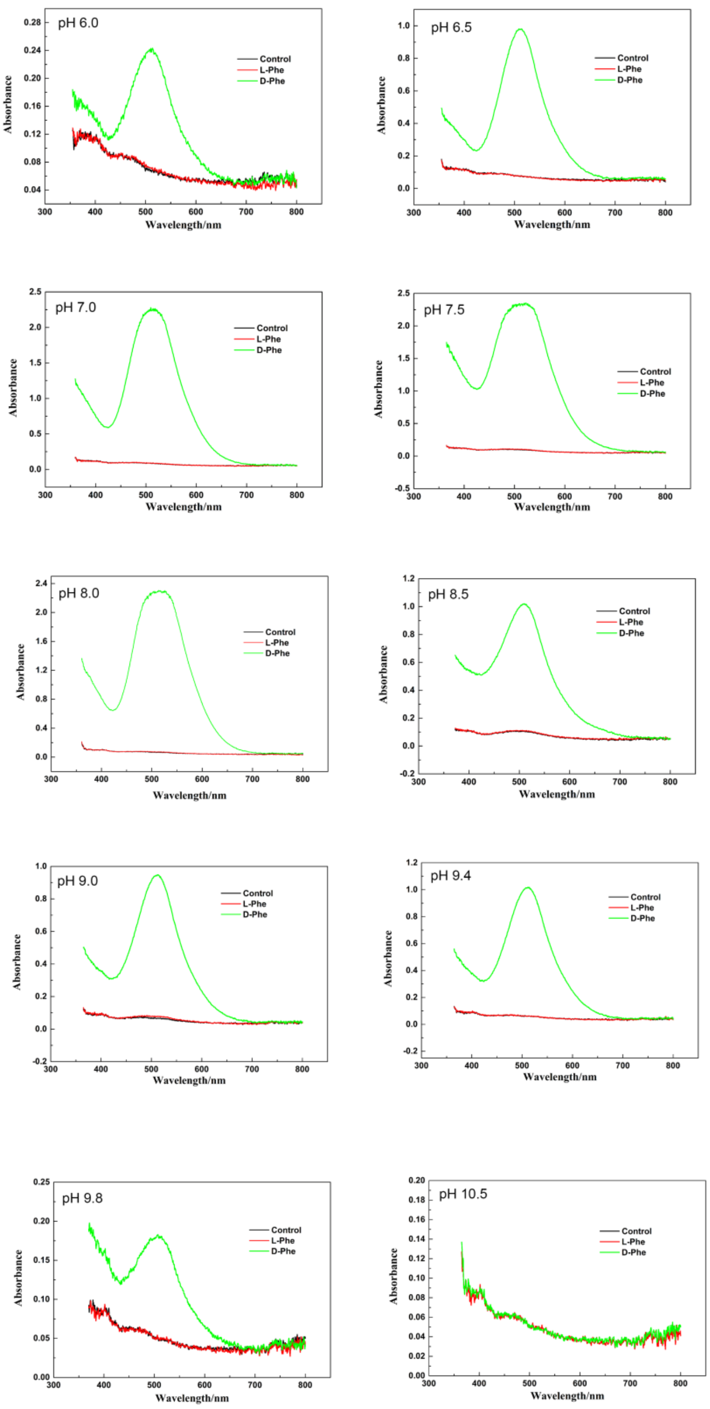

Figure S3 UV absorbance of UV-vis spectrum of the reaction product derived from DAAO and L-, D-Phe, the concentration of L-Phe and D-Phe was 5 mM.

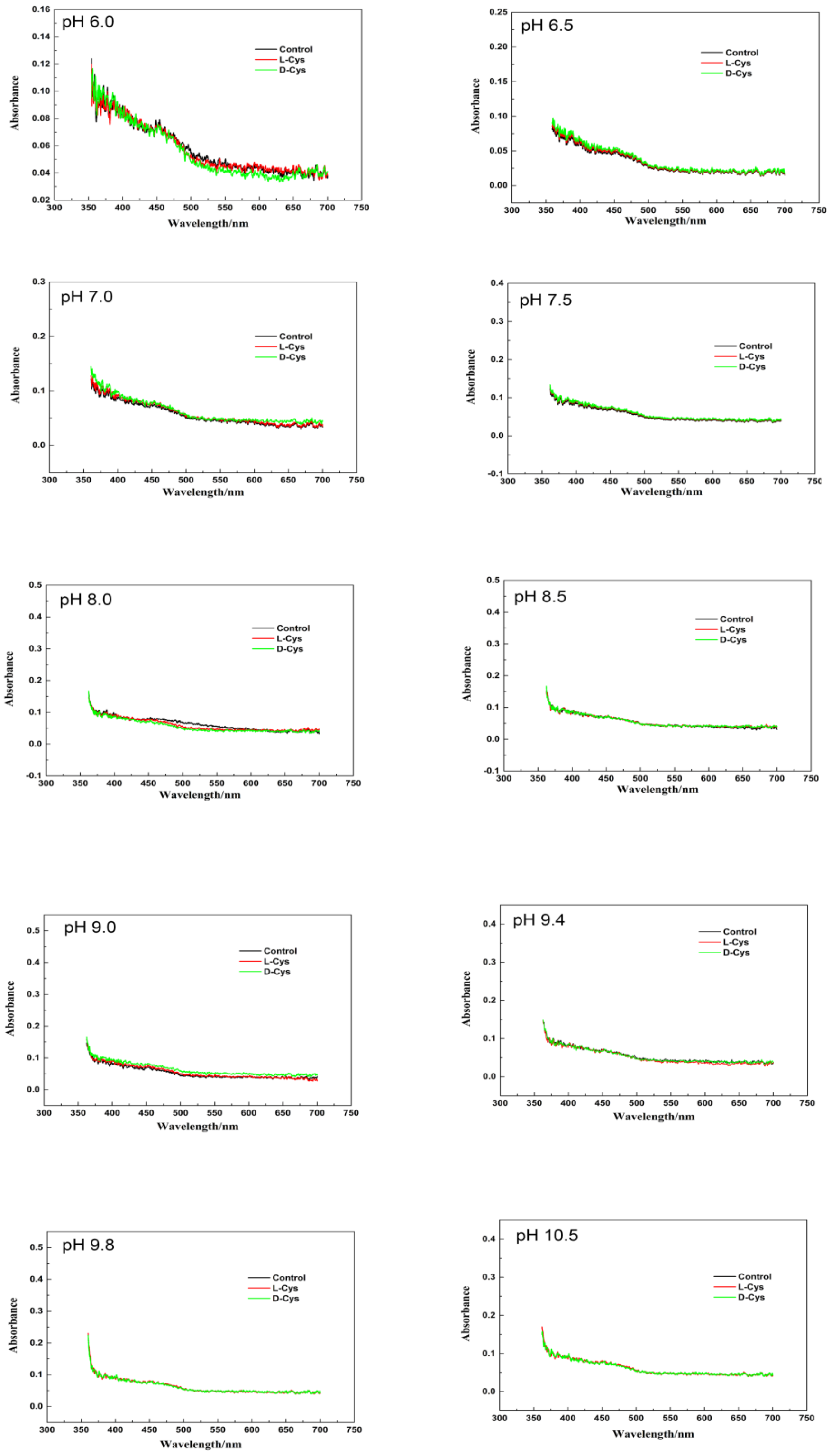

Figure S4 UV absorbance of UV-vis spectrum of the reaction product derived from DAAO and L-, D-Cys, the concentration of L-Cys and D-Cys was 5 mM.

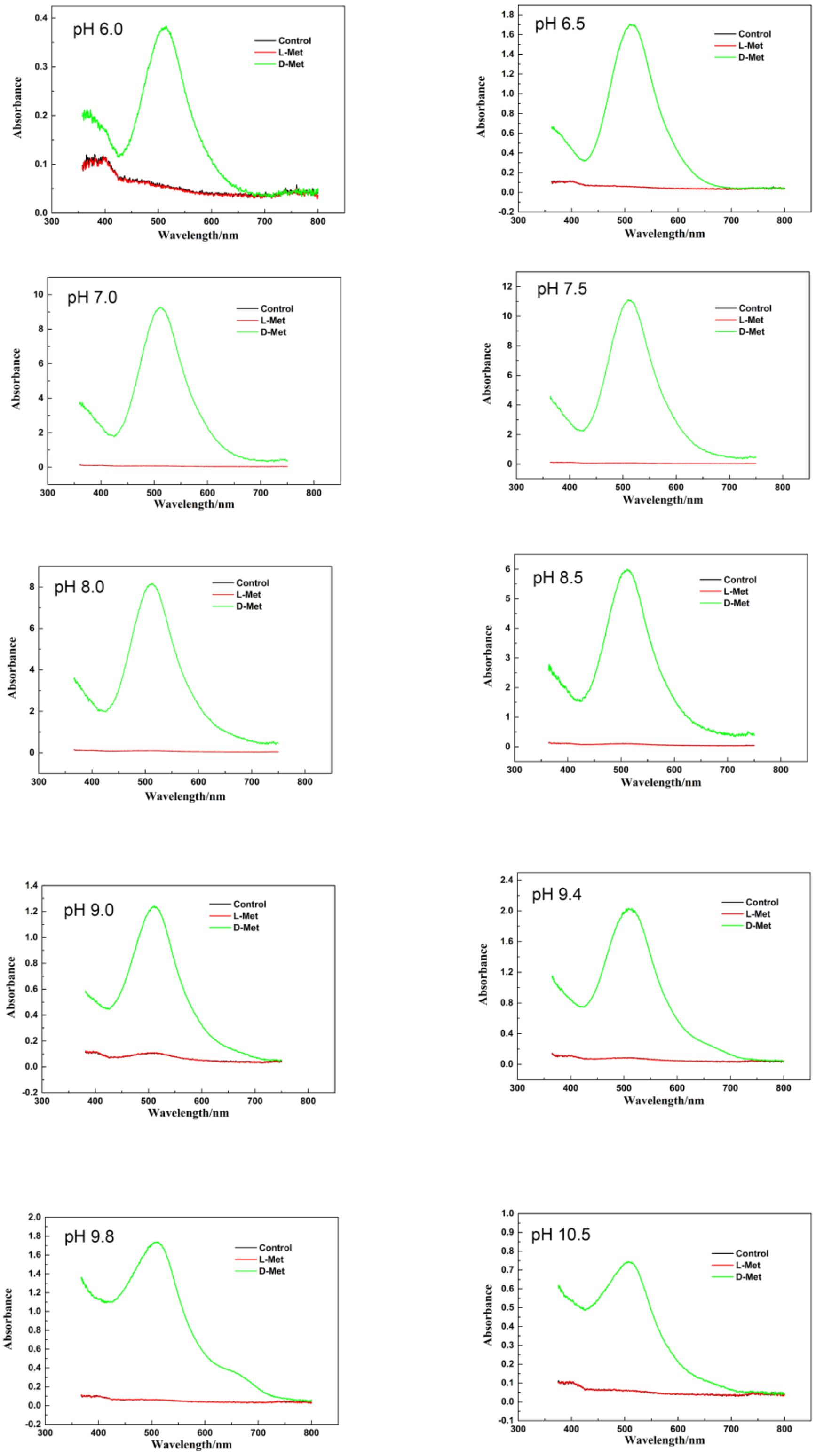

Figure S5 UV absorbance of UV-vis spectrum of the reaction product derived from DAAO and L-, D-Met, the concentration of L-Met and D-Met was 10 mM.

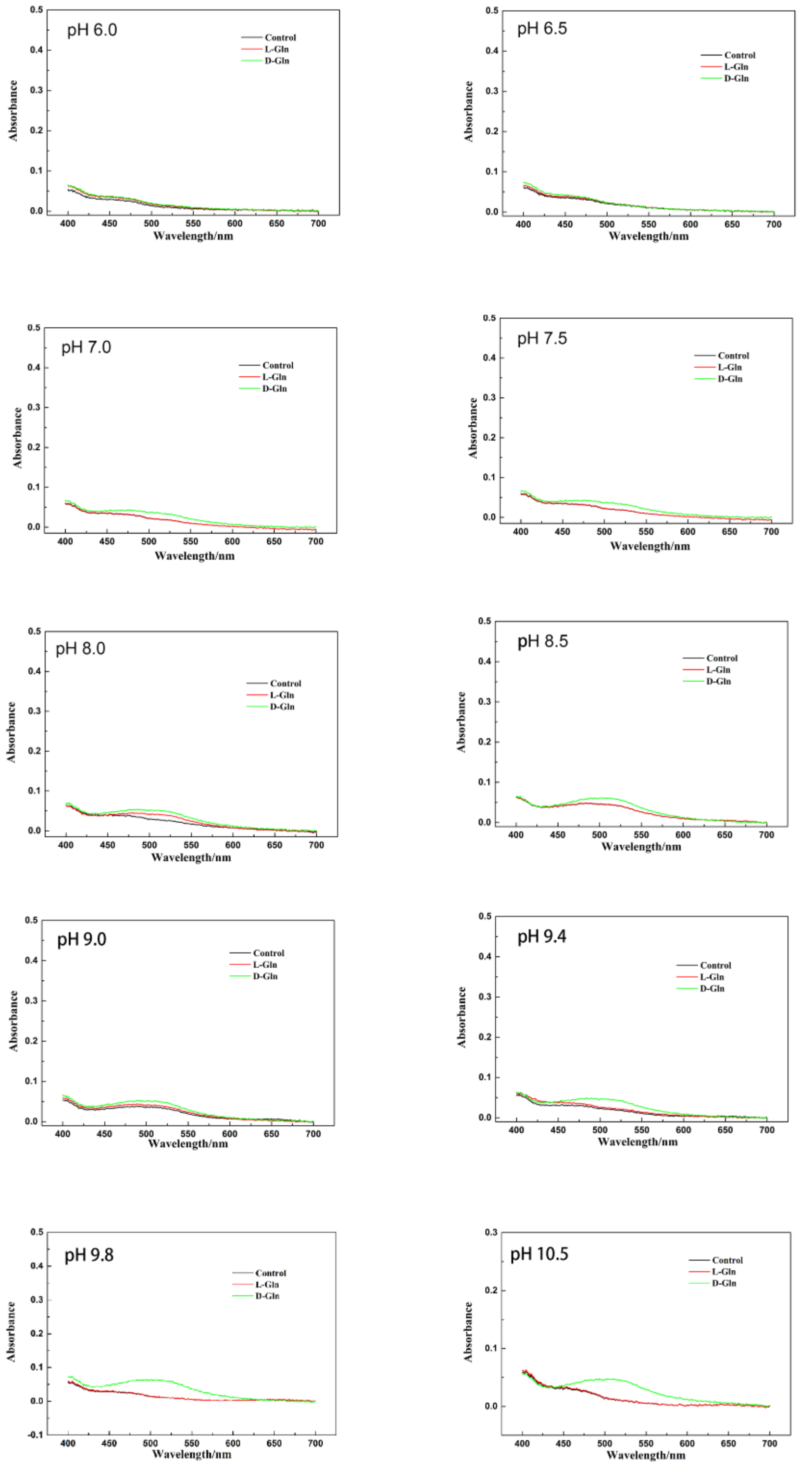

Figure S6 UV absorbance of UV-vis spectrum of the reaction product derived from DAAO and L-, D-Gln, the concentration of L-Gln and D-Gln was 5 mM.

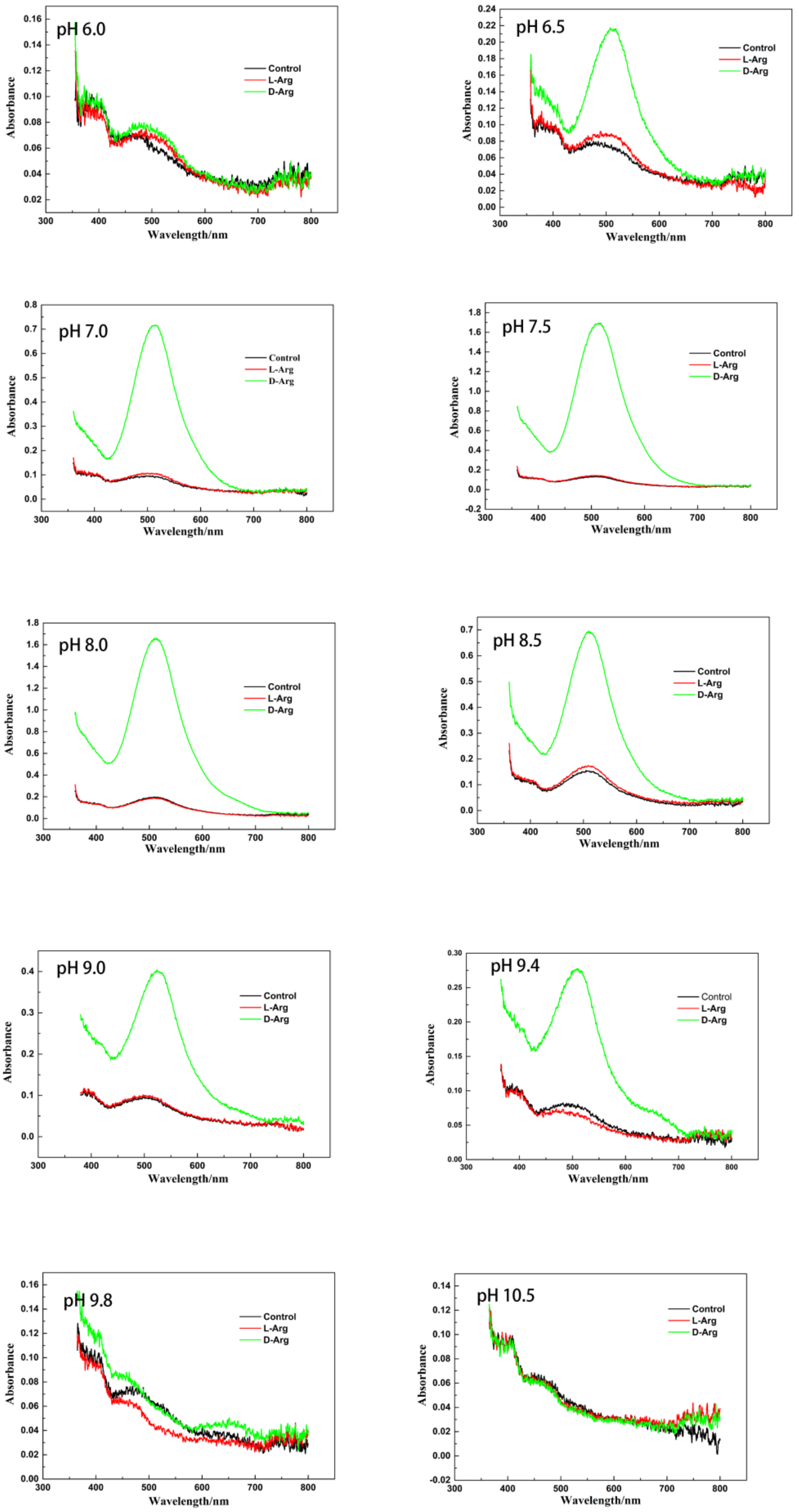

Figure S7 UV absorbance of UV-vis spectrum of the reaction product derived from DAAO and L-, D-Arg, the concentration of L-Arg and D-Arg was 10 mM.

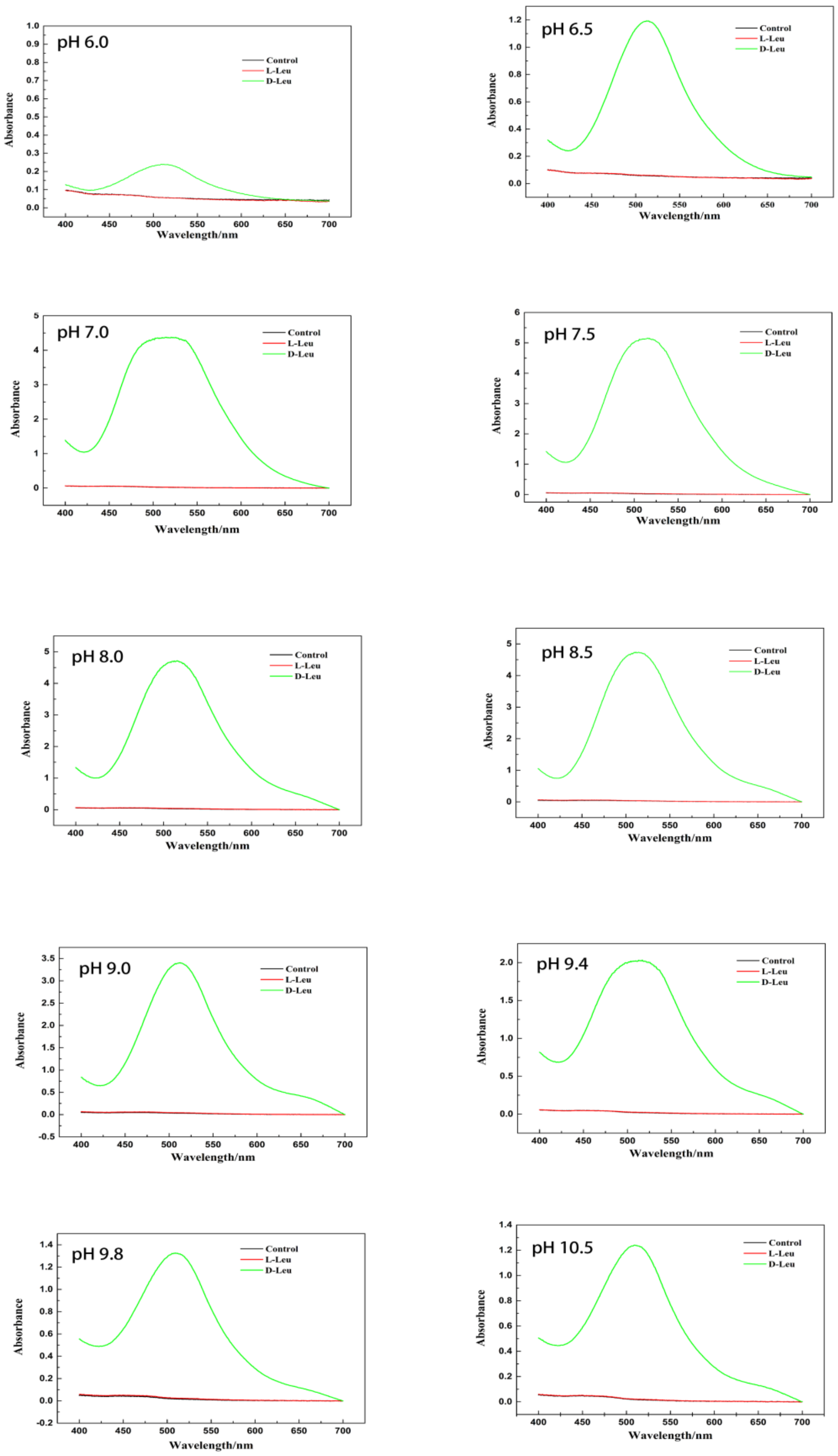

Figure S8 UV absorbance of UV-vis spectrum of the reaction product derived from DAAO and L-, D-Leu, the concentration of L-Leu and D-Leu was 18 mM.

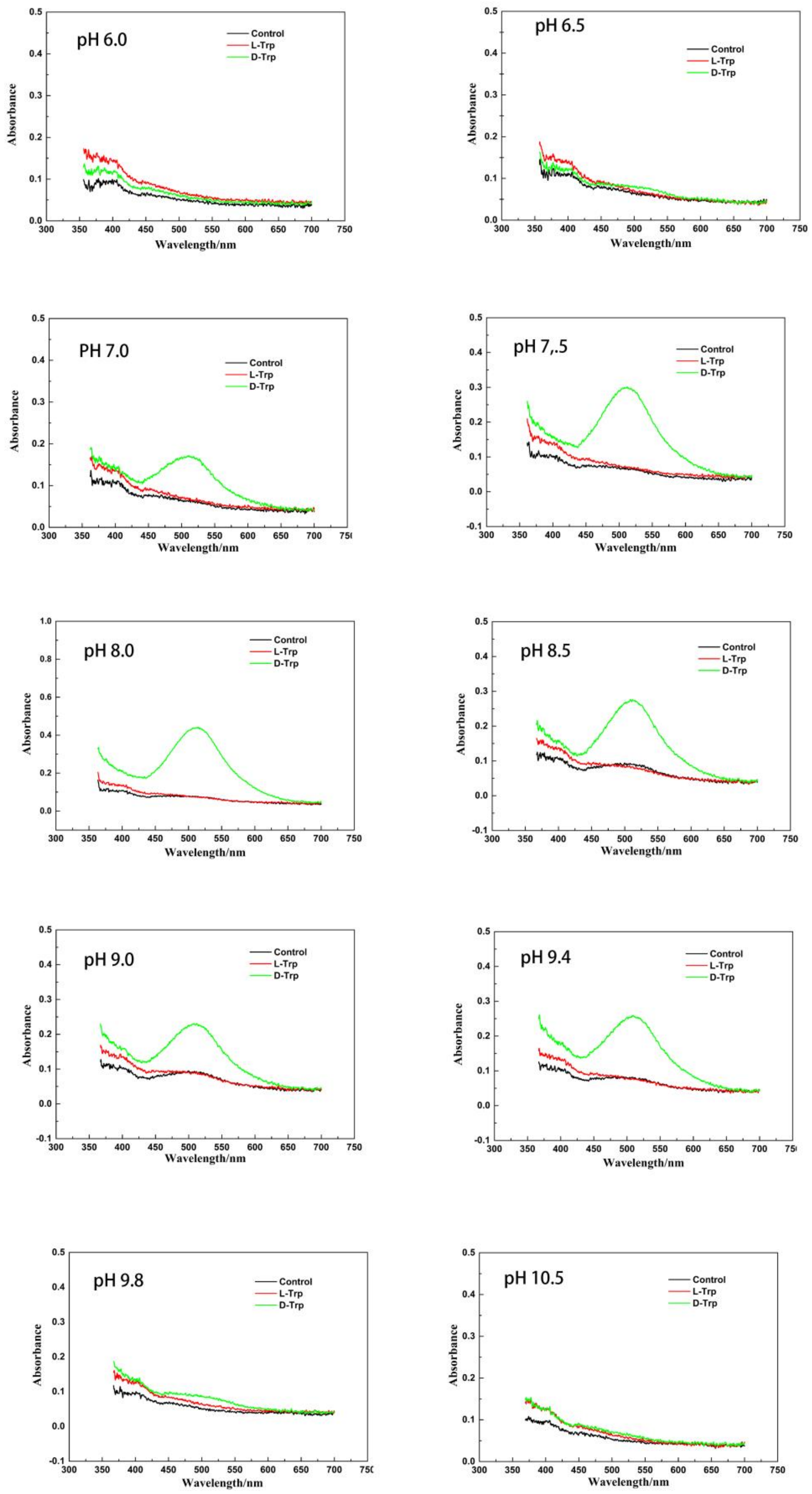

Figure S9 UV absorbance of UV-vis spectrum of the reaction product derived from DAAO and L-, D-Trp, the concentration of L-Trp and D-Trp was 5 mM.

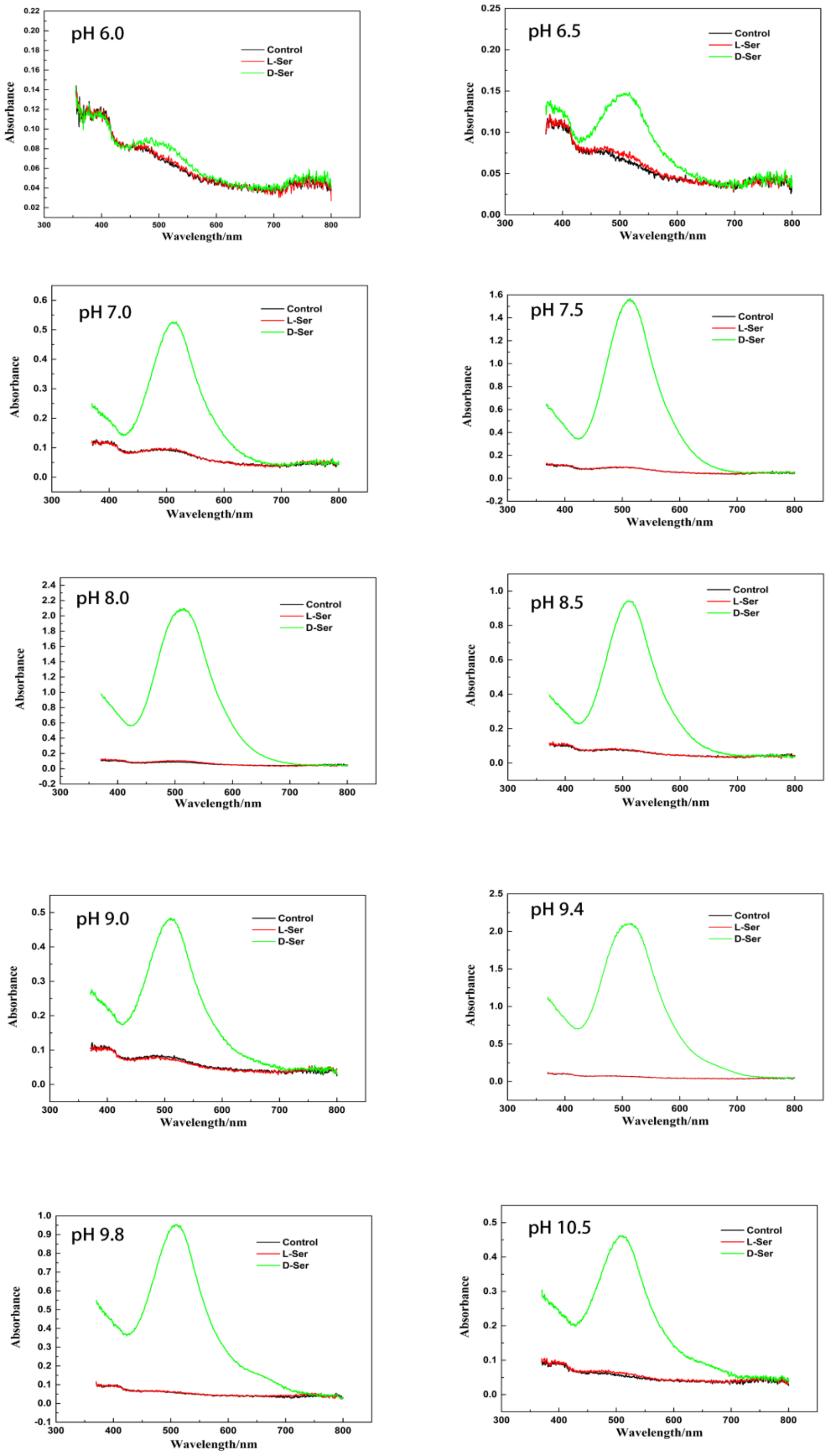

Figure S10 UV absorbance of UV-vis spectrum of the reaction product derived from DAAO and L-, D-Ser, the concentration of L-Ser and D-Ser was 5 mM.

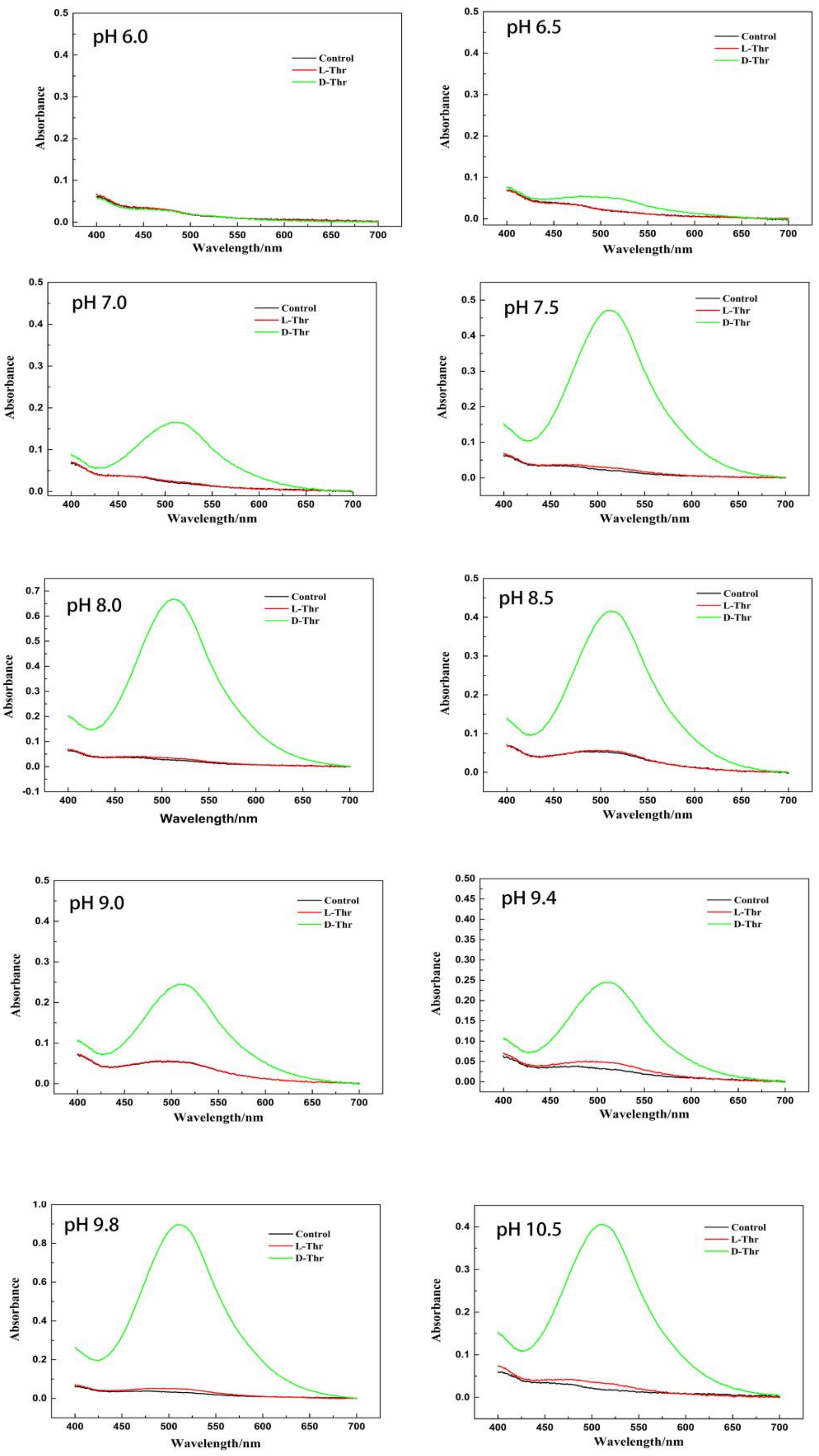

Figure S11 UV absorbance of UV-vis spectrum of the reaction product derived from DAAO and L-, D-Thr, the concentration of L-Thr and D-Thr was 5 mM.

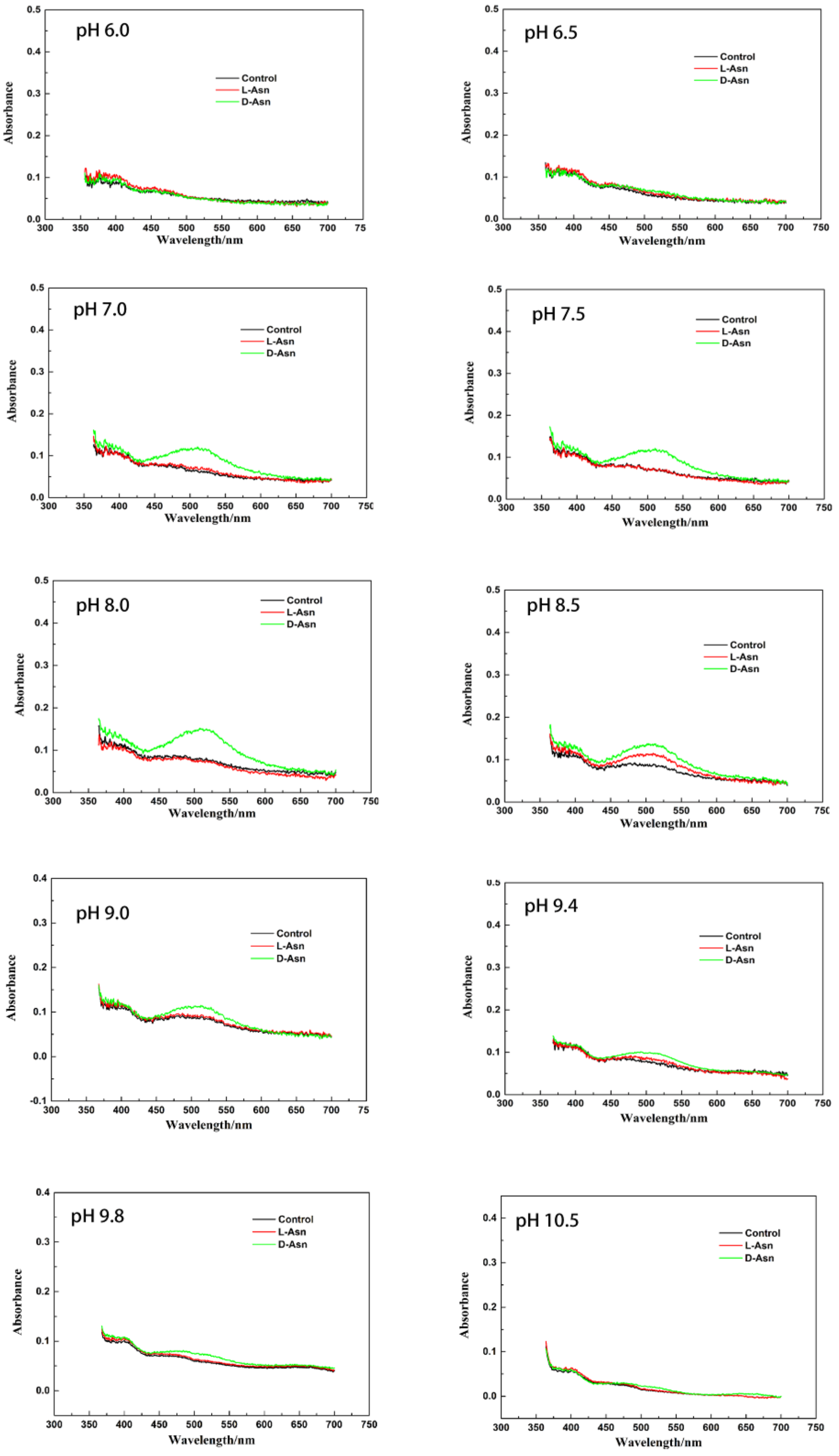

Figure S12 UV absorbance of UV-vis spectrum of the reaction product derived from DAAO and L-, D-Asn, the concentration of L-Asn and D-Asn was 10 mM.

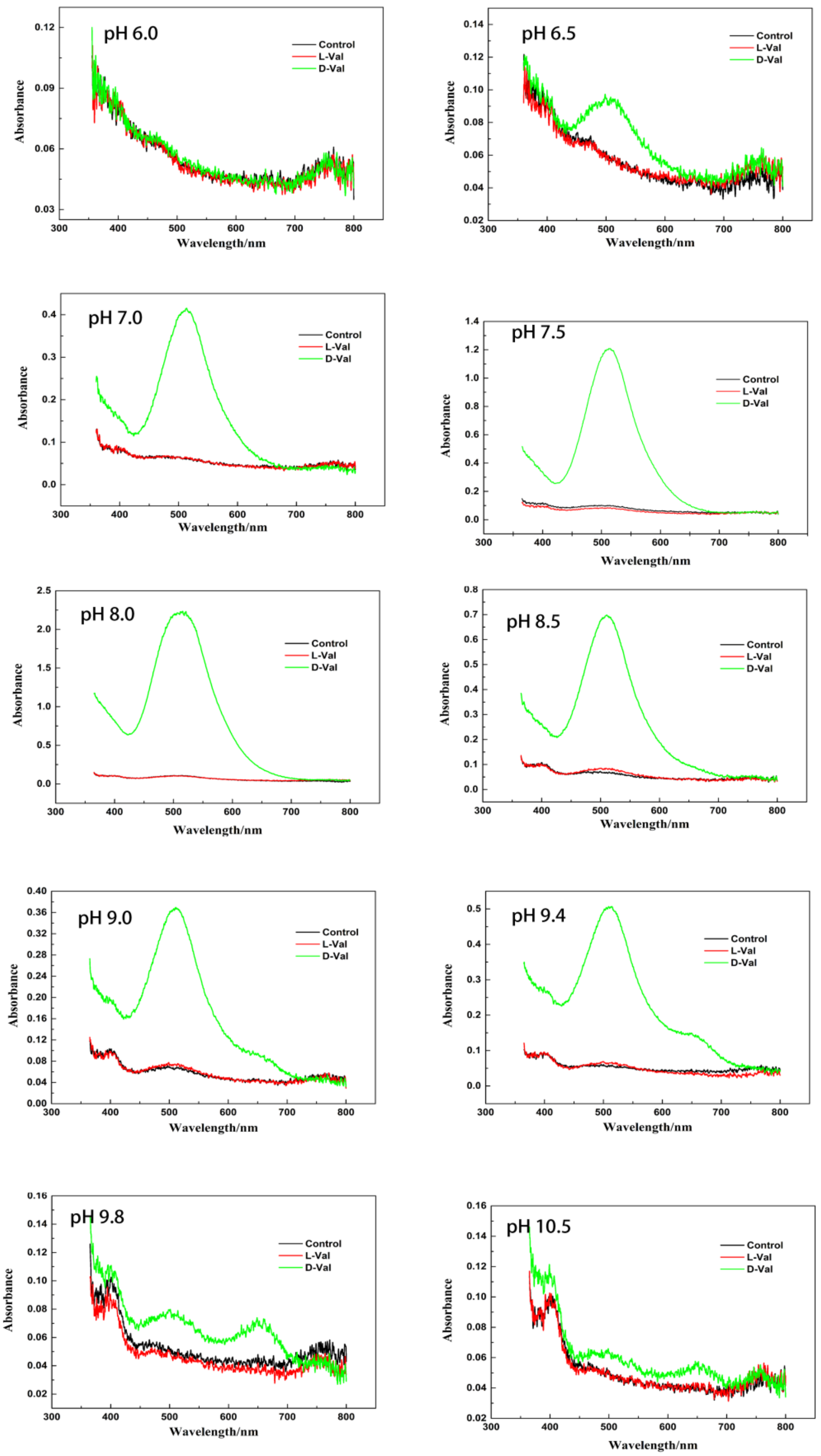

Figure S13 UV absorbance of UV-vis spectrum of the reaction product derived from DAAO and L-, D-Val, the concentration of L-Val and D-Val was 10 mM.

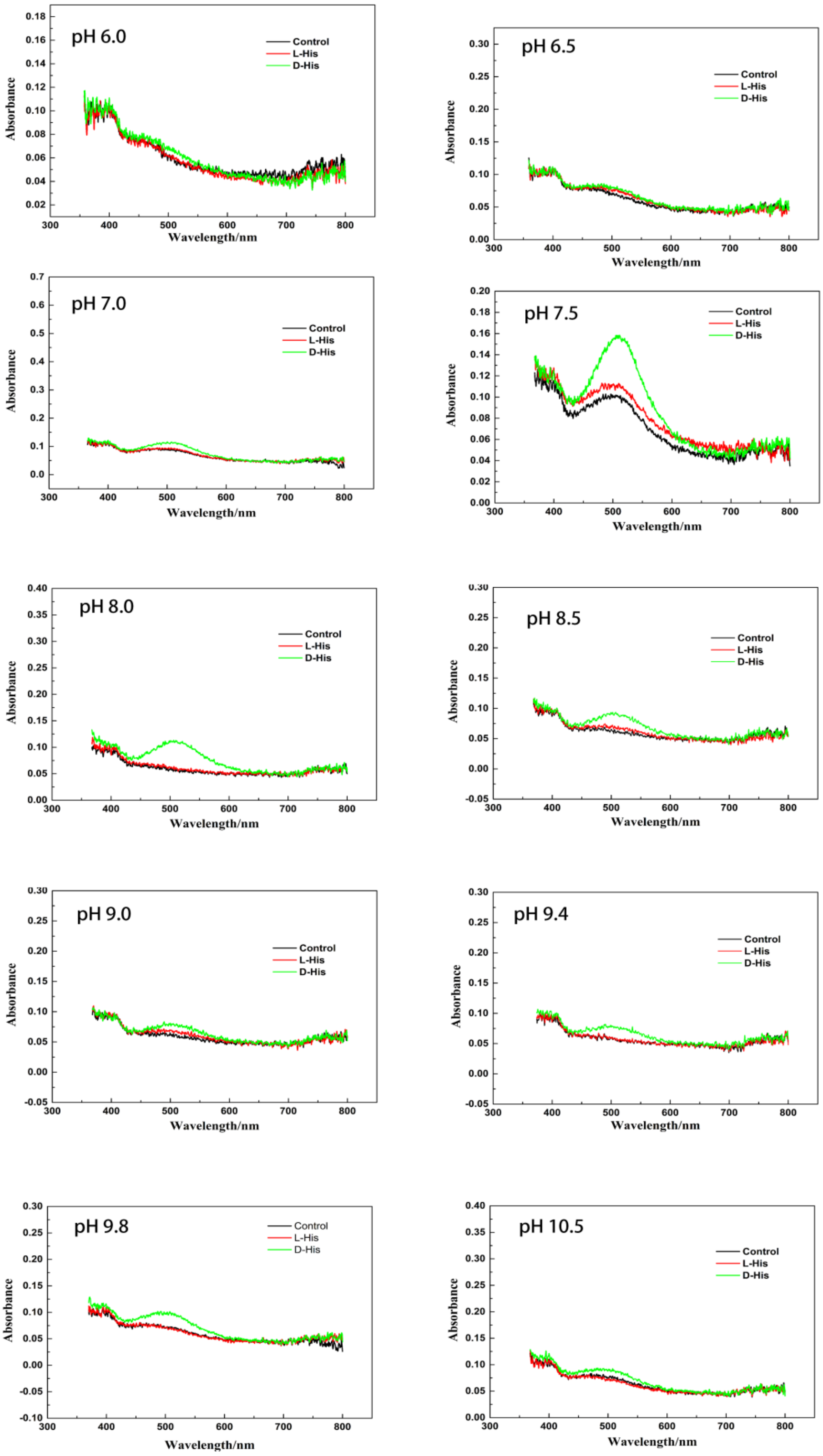

Figure S14 UV absorbance of UV-vis spectrum of the reaction product derived from DAAO and L-, D-His, the concentration of L-His and D-His was 10 mM.

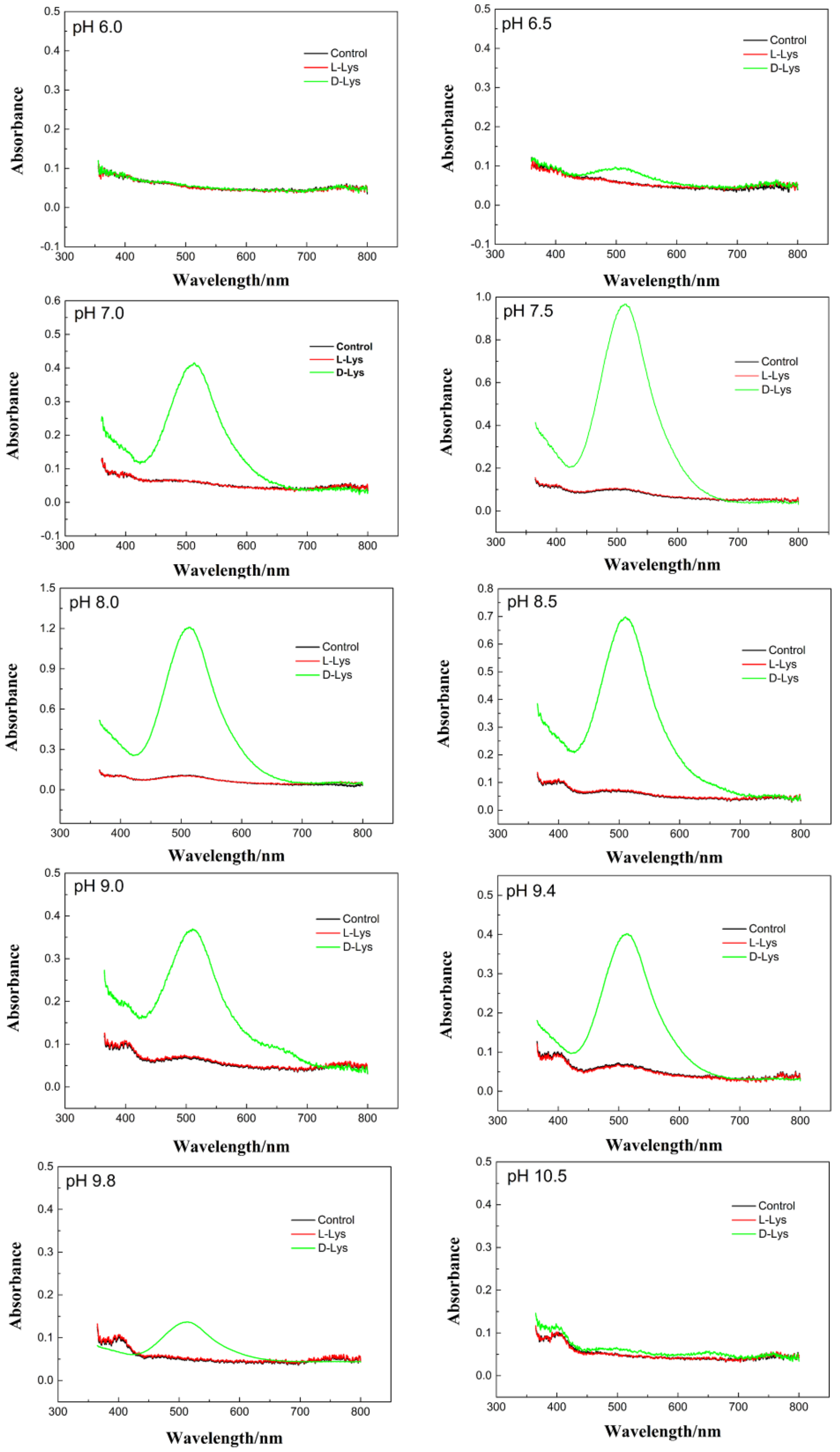

Figure S15 UV absorbance of UV-vis spectrum of the reaction product derived from DAAO and L-, D-Lys, the concentration of L-Lys and D-Lys was 10 mM.

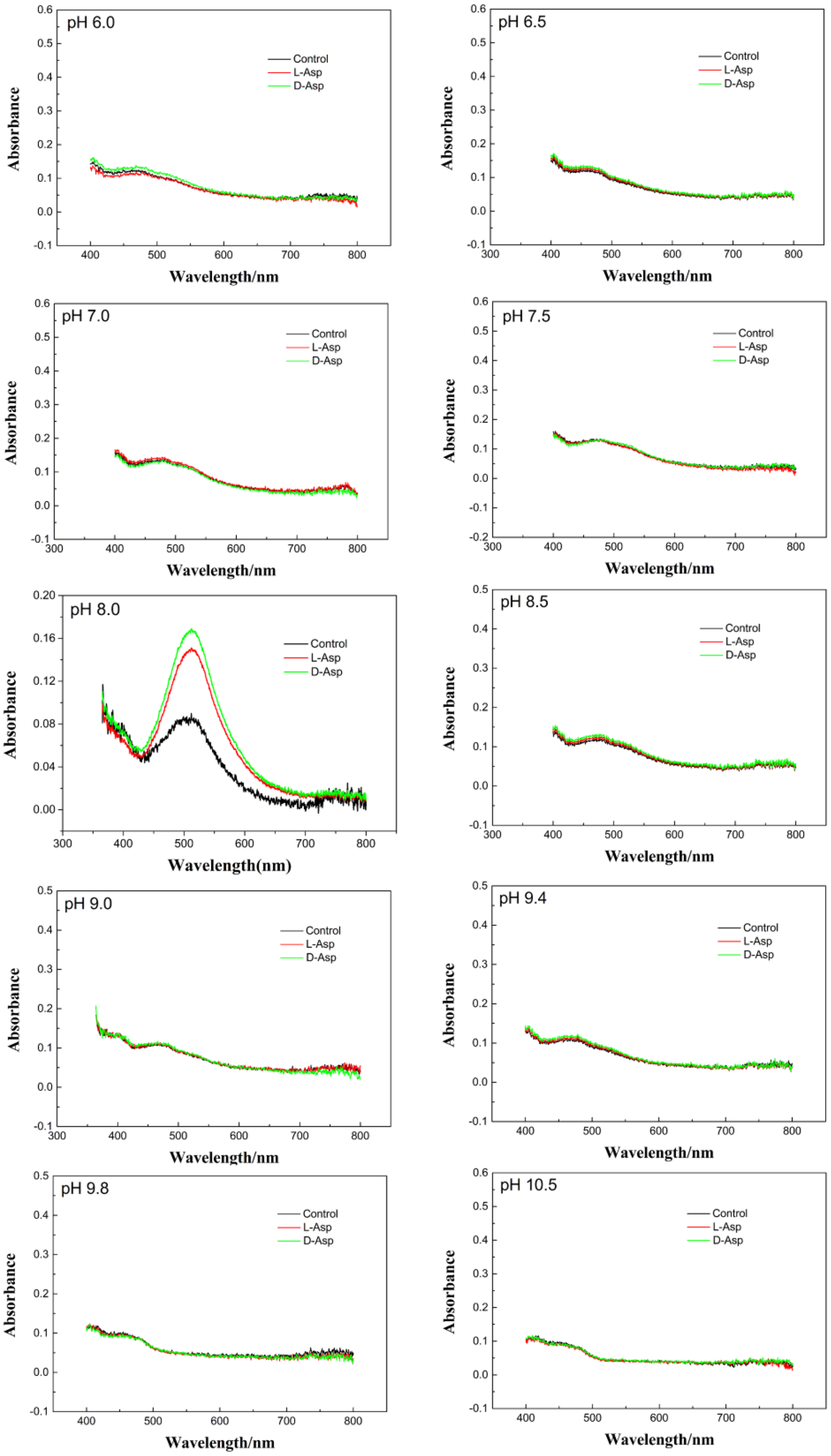

Figure S16 UV absorbance of UV-vis spectrum of the reaction product derived from DAAO and L-, D-Asp, the concentration of L-Asp and D-Asp was 1 mM.

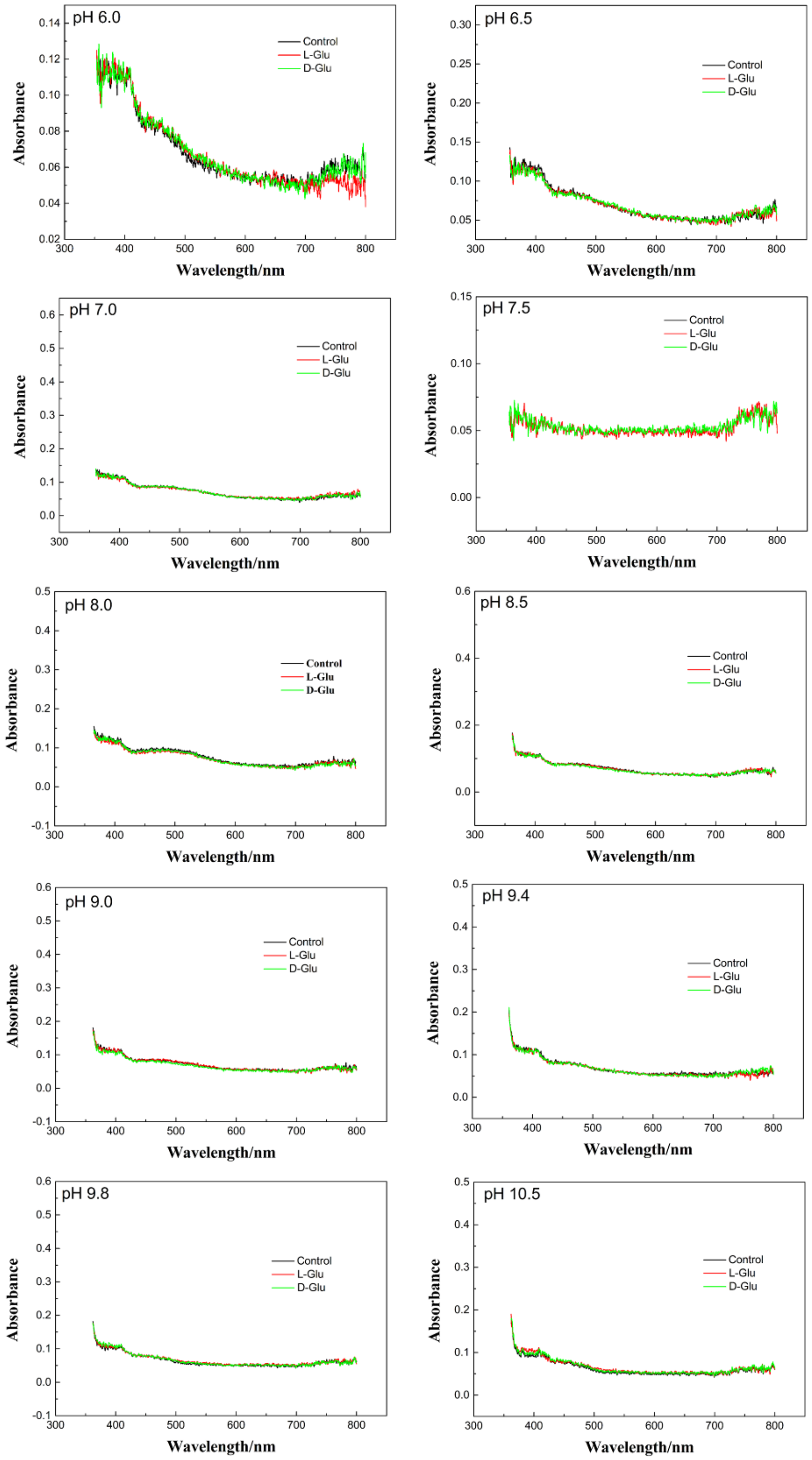

Figure S17 UV absorbance of UV-vis spectrum of the reaction product derived from DAAO and L-, D-Glu, the concentration of L-Glu and D-Glu was 1 mM.

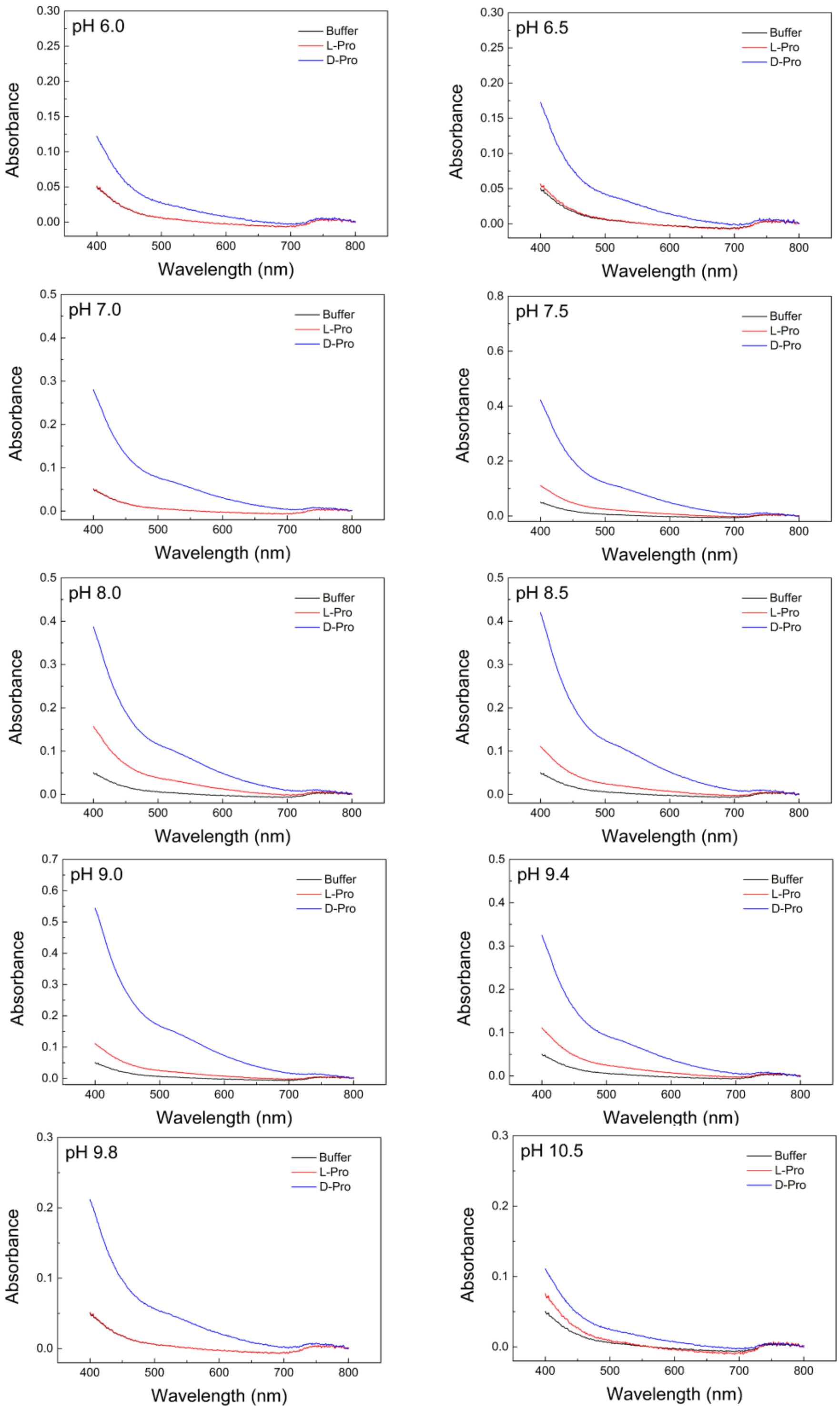

Figure S18 UV absorbance of UV-vis spectrum of the reaction product derived from DAAO and L-, D-Pro, the concentration of L-Pro and D-Pro was 25 mM. The reactants derived from D-Pro were diluted into one fifth and determined.

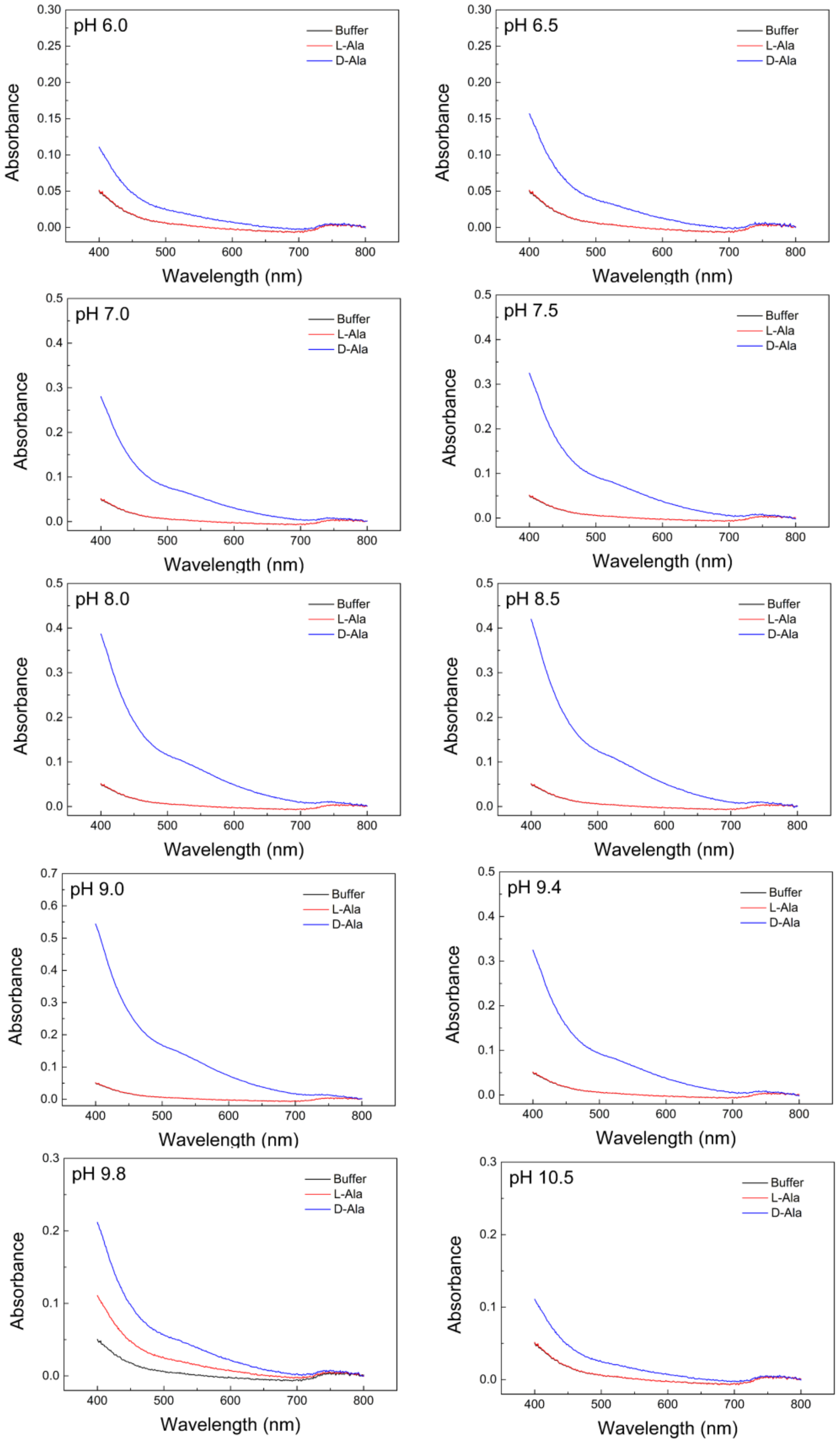

Figure S19 UV absorbance of UV-vis spectrum of the reaction product derived from DAAO and L-, D-Ala, the concentration of L-Ala and D-Ala was 25 mM. The reactants derived from D-Ala were diluted into one fifth and determined.

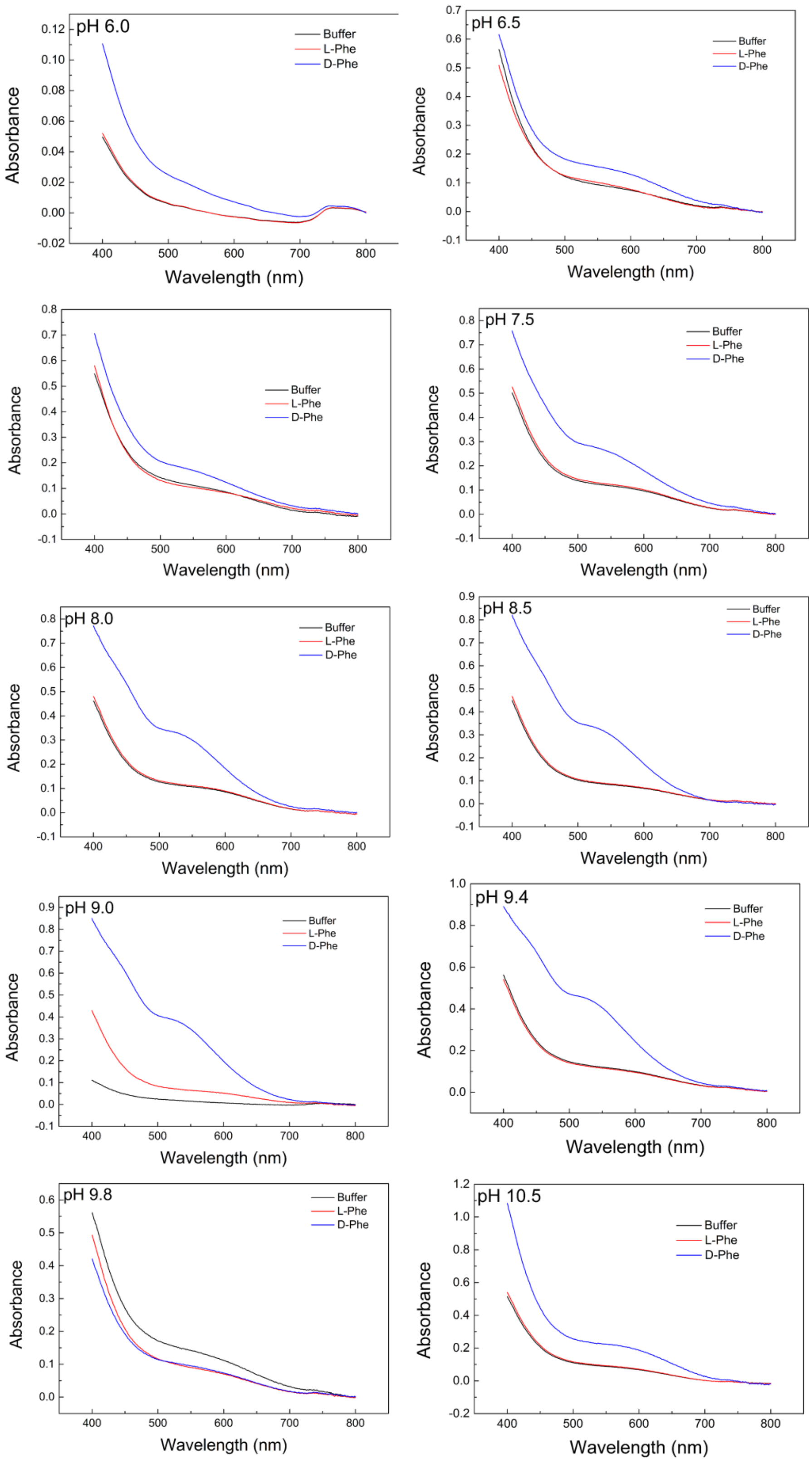

Figure S20 UV absorbance of UV-vis spectrum of the reaction product derived from DAAO and L-, D-Phe, the concentration of L-Phe and D-Phe was 10 mM. The reactants derived from D-Phe were diluted into twice and determined.

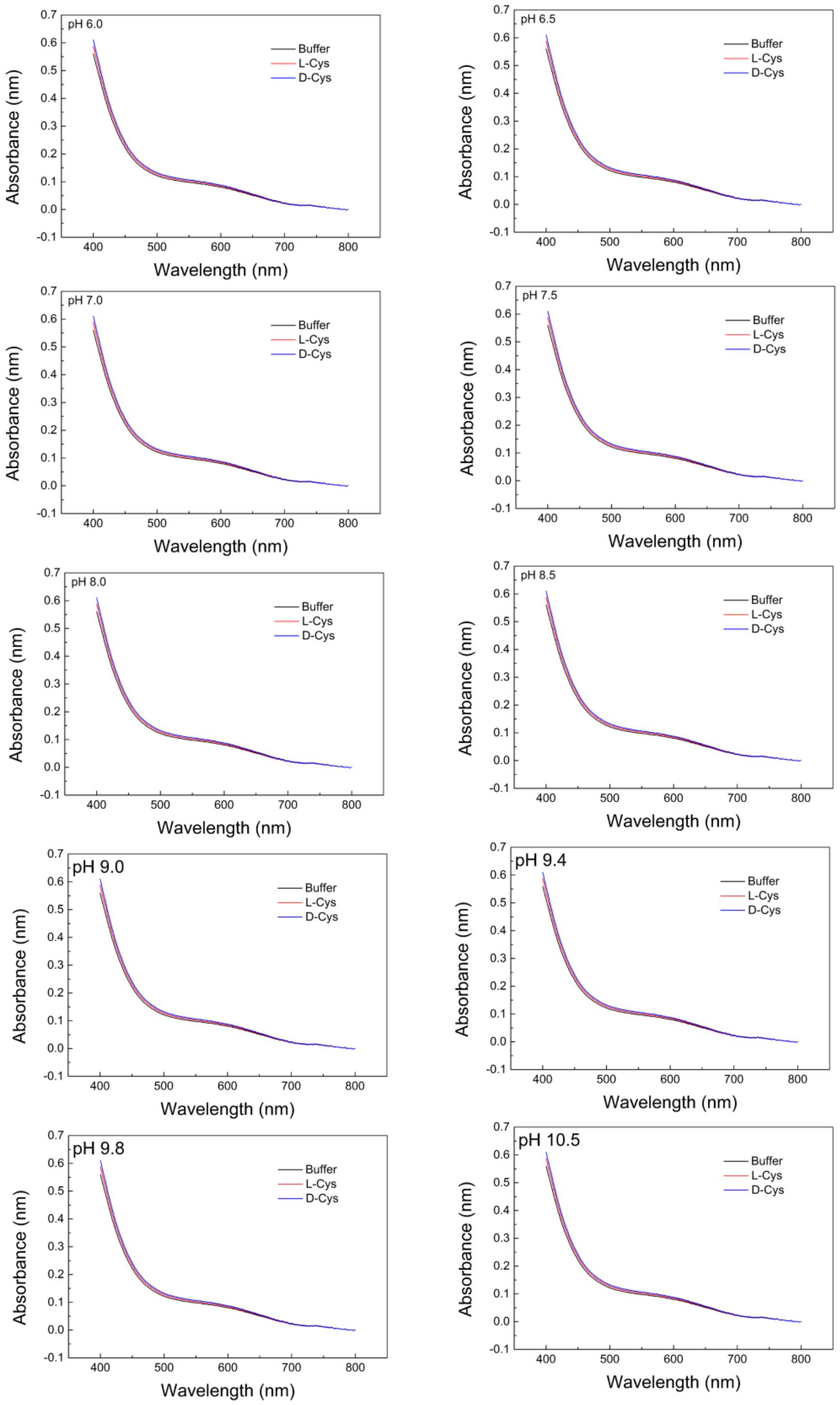

Figure S21 UV absorbance of UV-vis spectrum of the reaction product derived from DAAO and L-, D-Cys, the concentration of L-Cys and D-Cys was 5 mM.

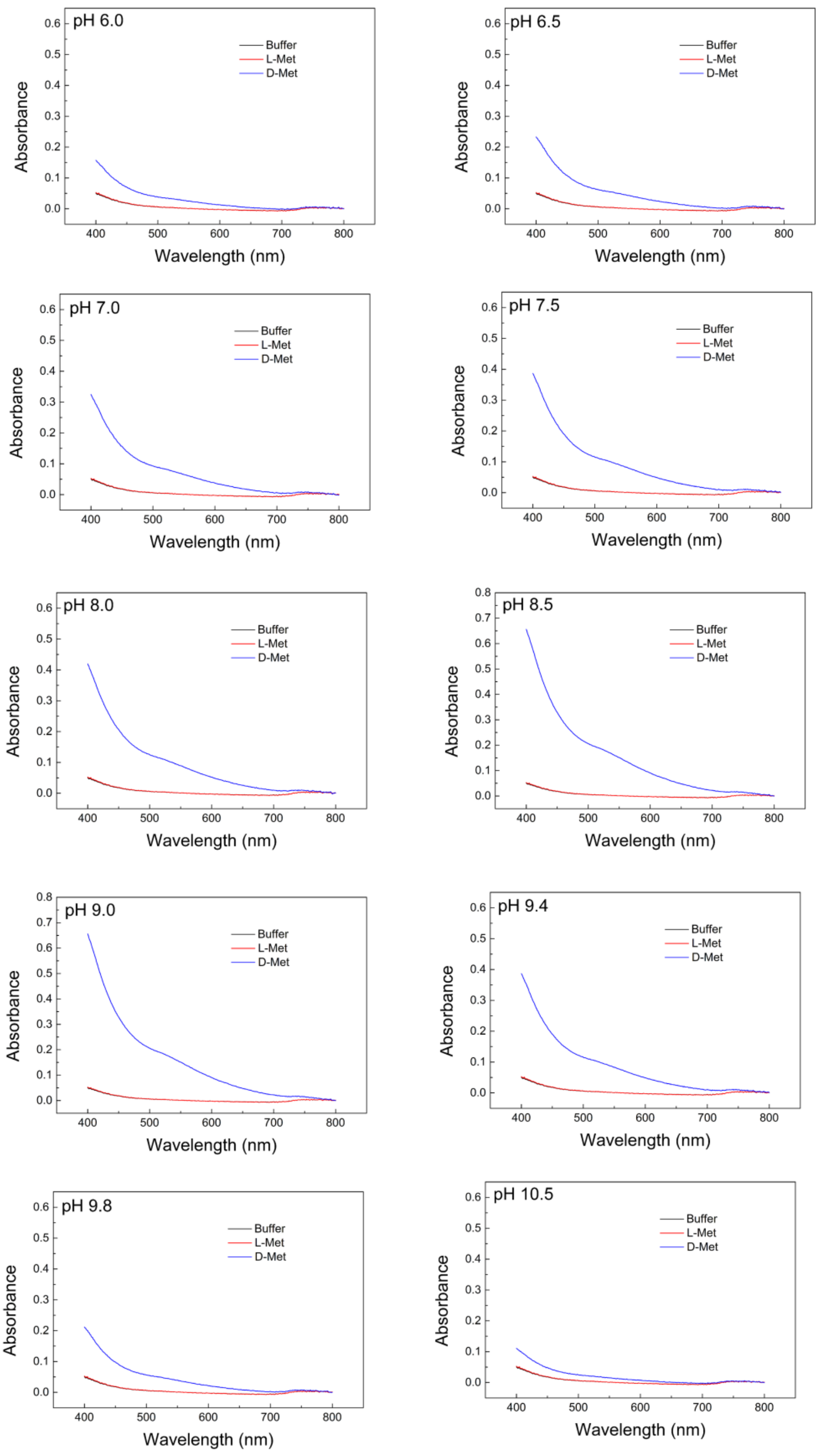

Figure S22 UV absorbance of UV-vis spectrum of the reaction product derived from DAAO and L-, D-Met, the concentration of L-Met and D-Met was 10 mM. The reactants derived from D-Met were diluted into fifth and determined.

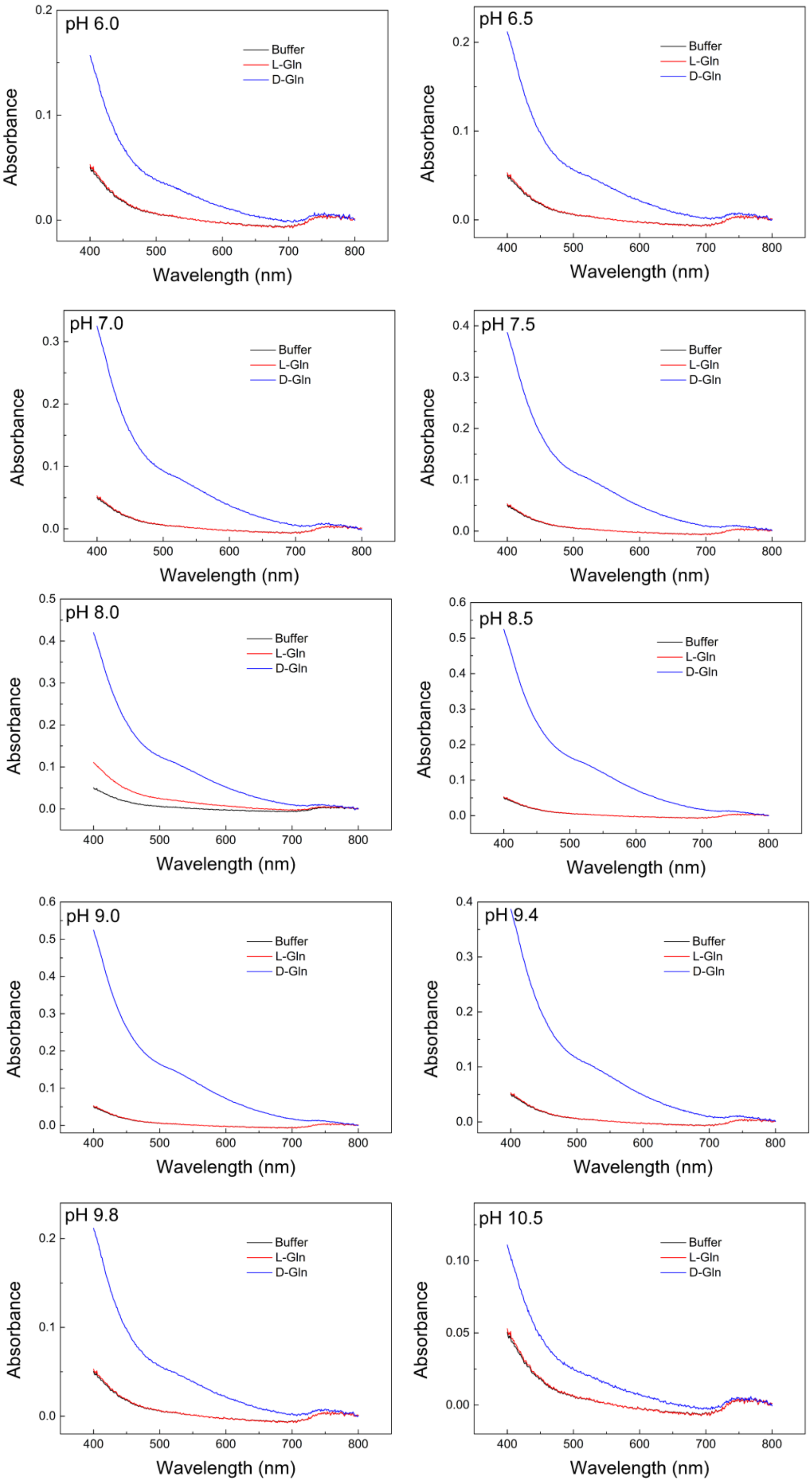

Figure S23 UV absorbance of UV-vis spectrum of the reaction product derived from DAAO and L-, D-Gln, the concentration of L-Gln and D-Gln was 5 mM.

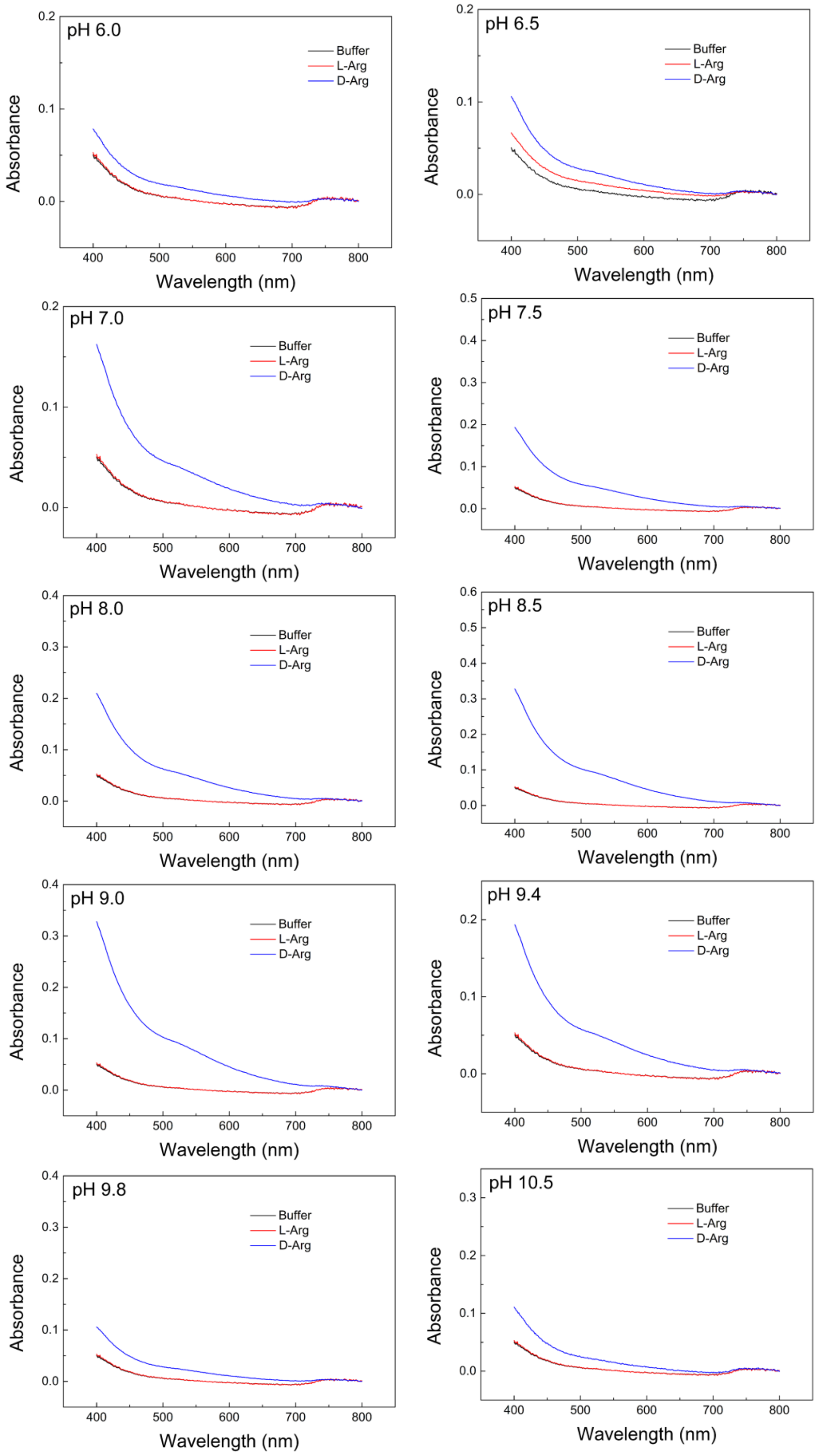

Figure S24 UV absorbance of UV-vis spectrum of the reaction product derived from DAAO and L-, D-Arg, the concentration of L-Arg and D-Arg was 2 mM. The reactants derived from D-Arg were diluted into twice and determined.

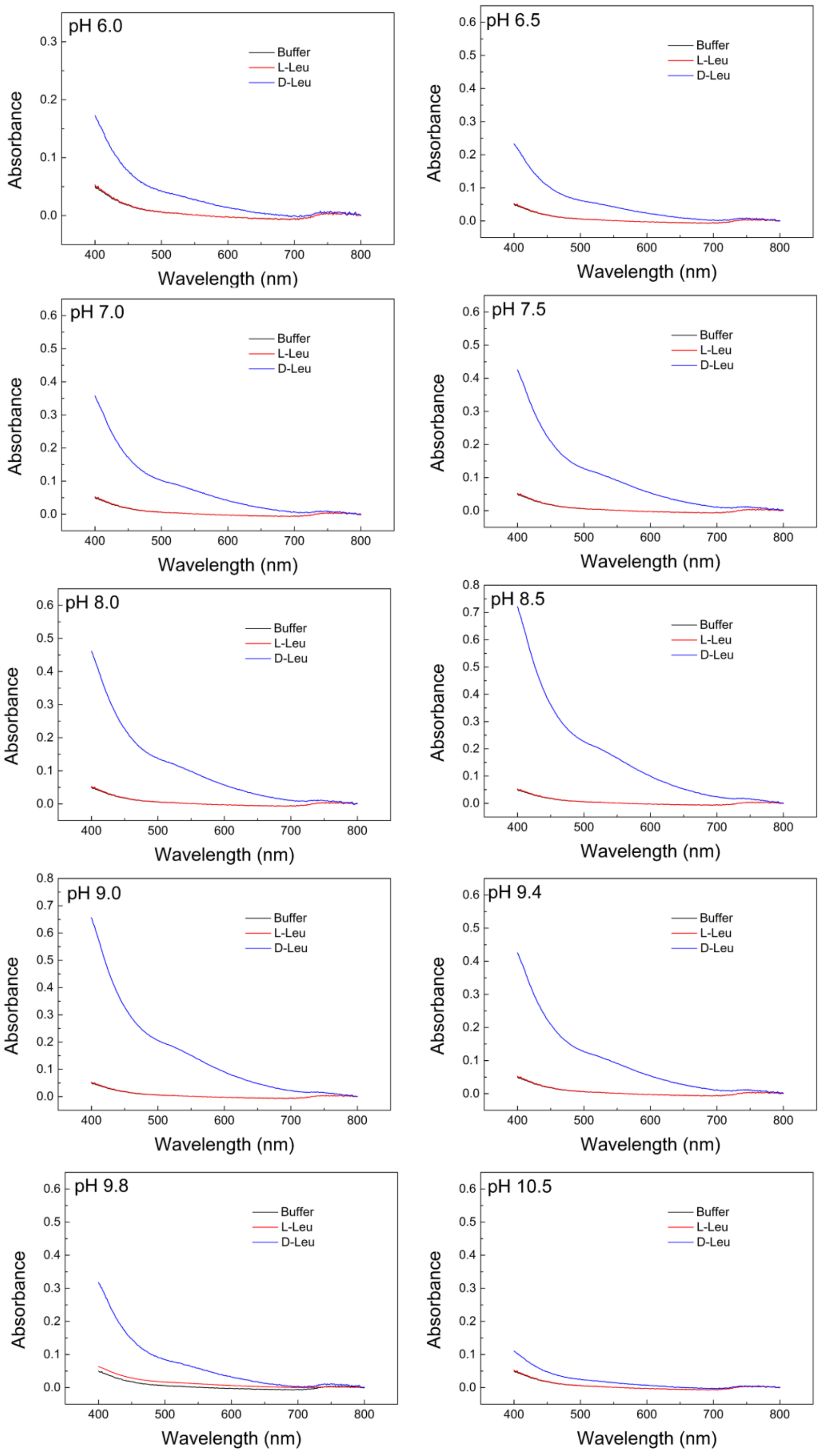

Figure S25 UV absorbance of UV-vis spectrum of the reaction product derived from DAAO and L-, D-Leu, the concentration of L-Leu and D-Leu was 18 mM.

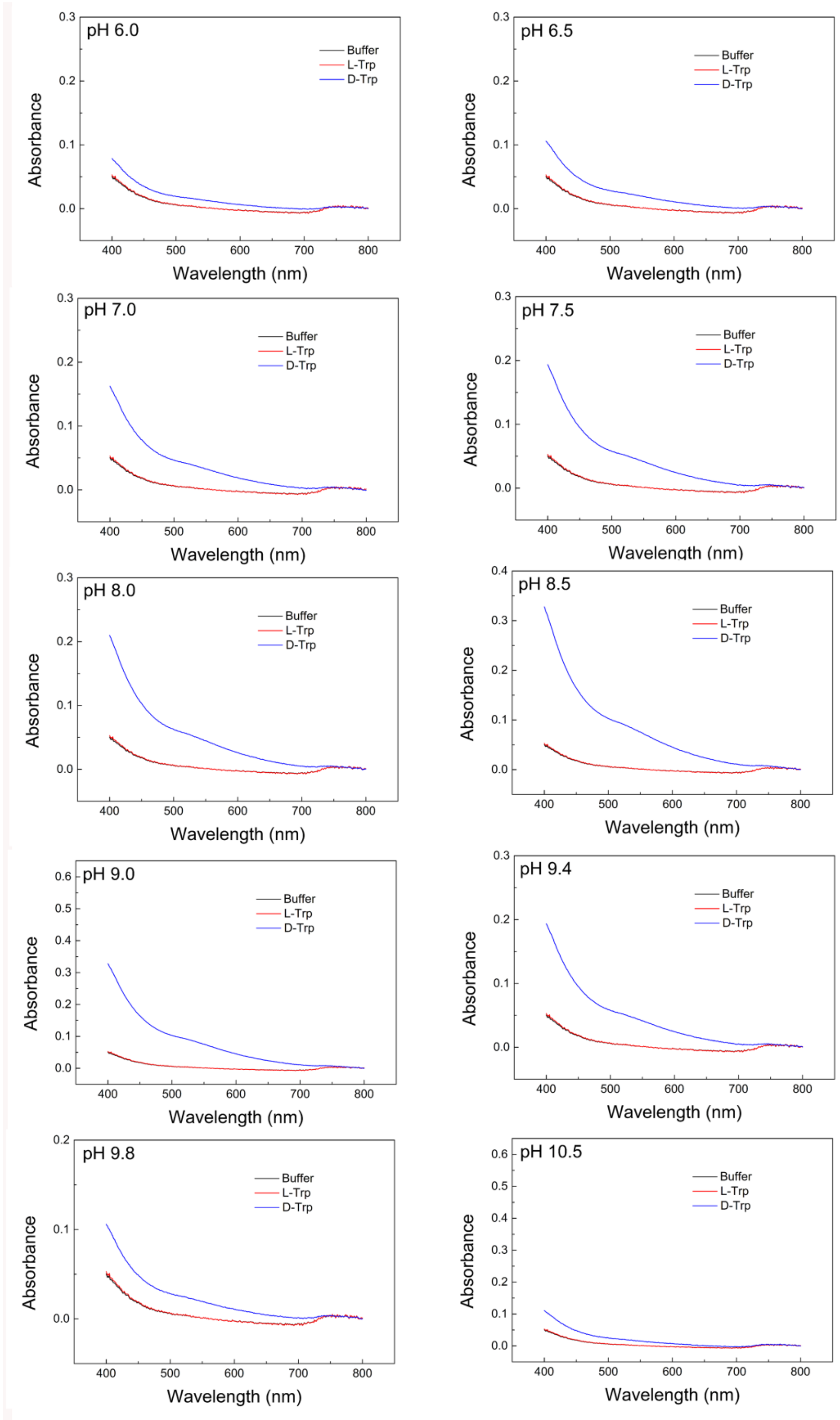

Figure S26 UV absorbance of UV-vis spectrum of the reaction product derived from DAAO and L-, D-Trp, the concentration of L-Trp and D-Trp was 5 mM.

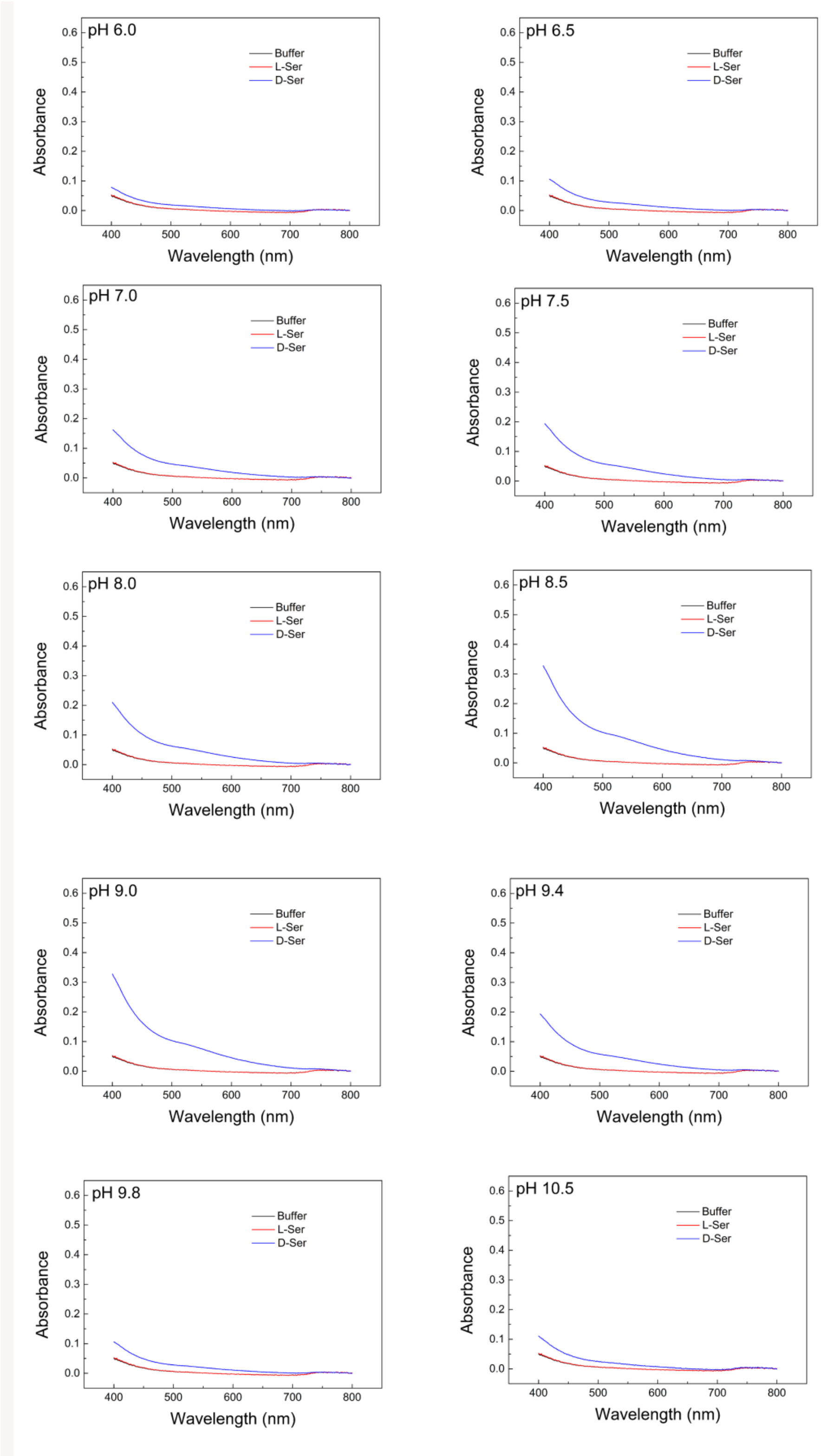

Figure S27 UV absorbance of UV-vis spectrum of the reaction product derived from DAAO and L-, D-Ser, the concentration of L-Ser and D-Ser was 5 mM.

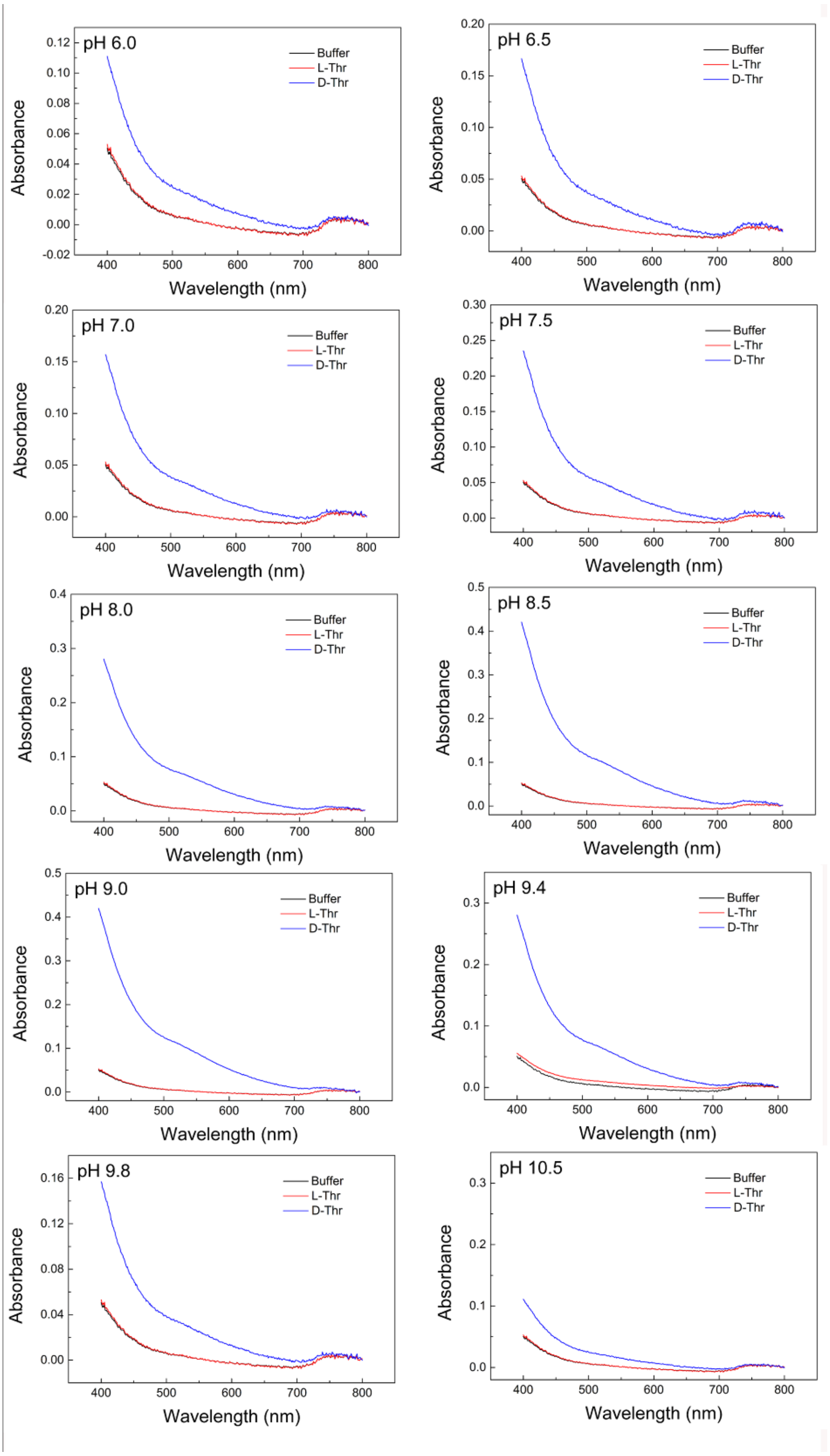

Figure S28 UV absorbance of UV-vis spectrum of the reaction product derived from DAAO and L-, D-Thr, the concentration of L-Thr and D-Thr was 5 mM.

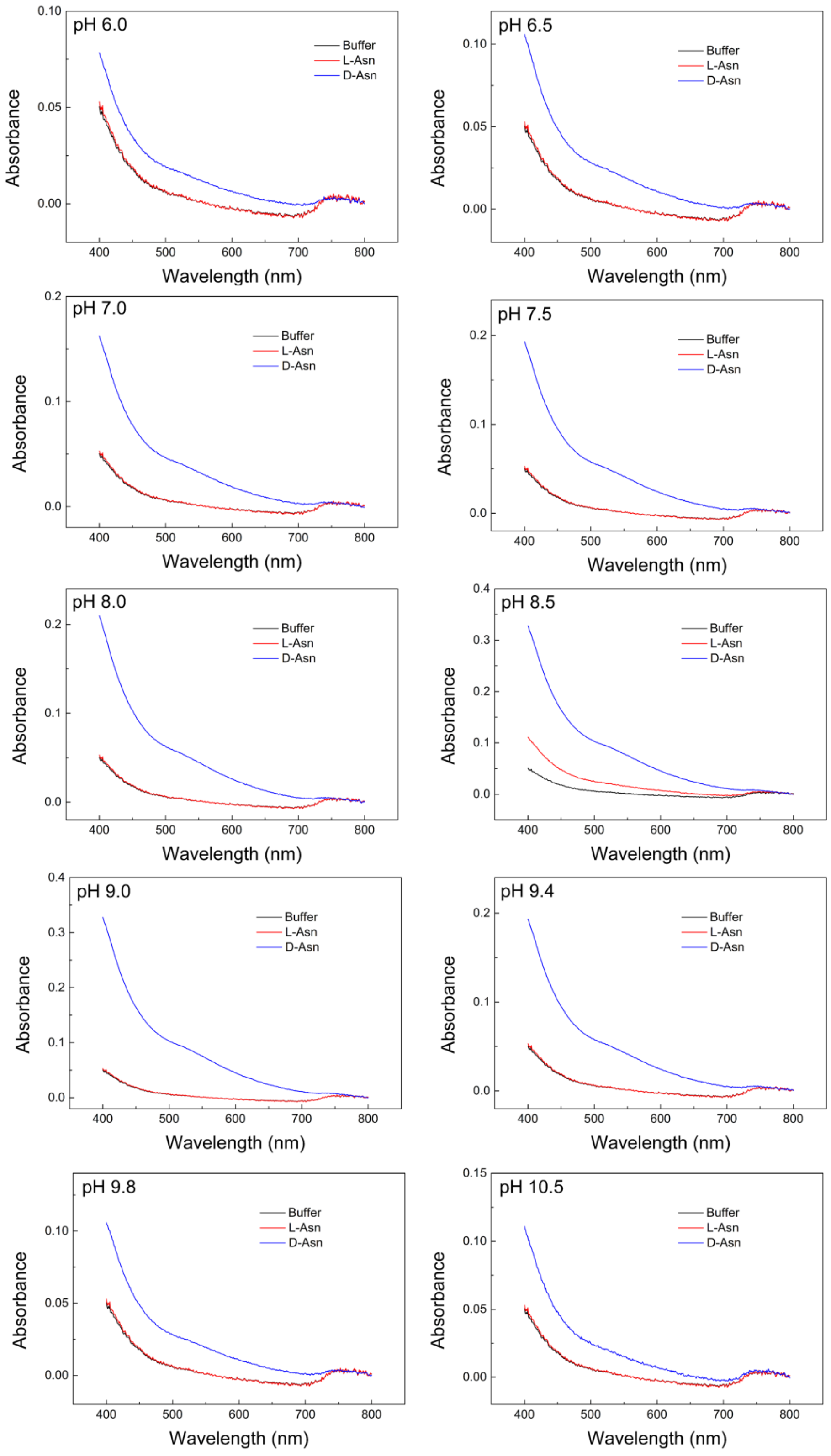

Figure S29 UV absorbance of UV-vis spectrum of the reaction product derived from DAAO and L-, D-Asn, the concentration of L-Asn and D-Asn was 10 mM.

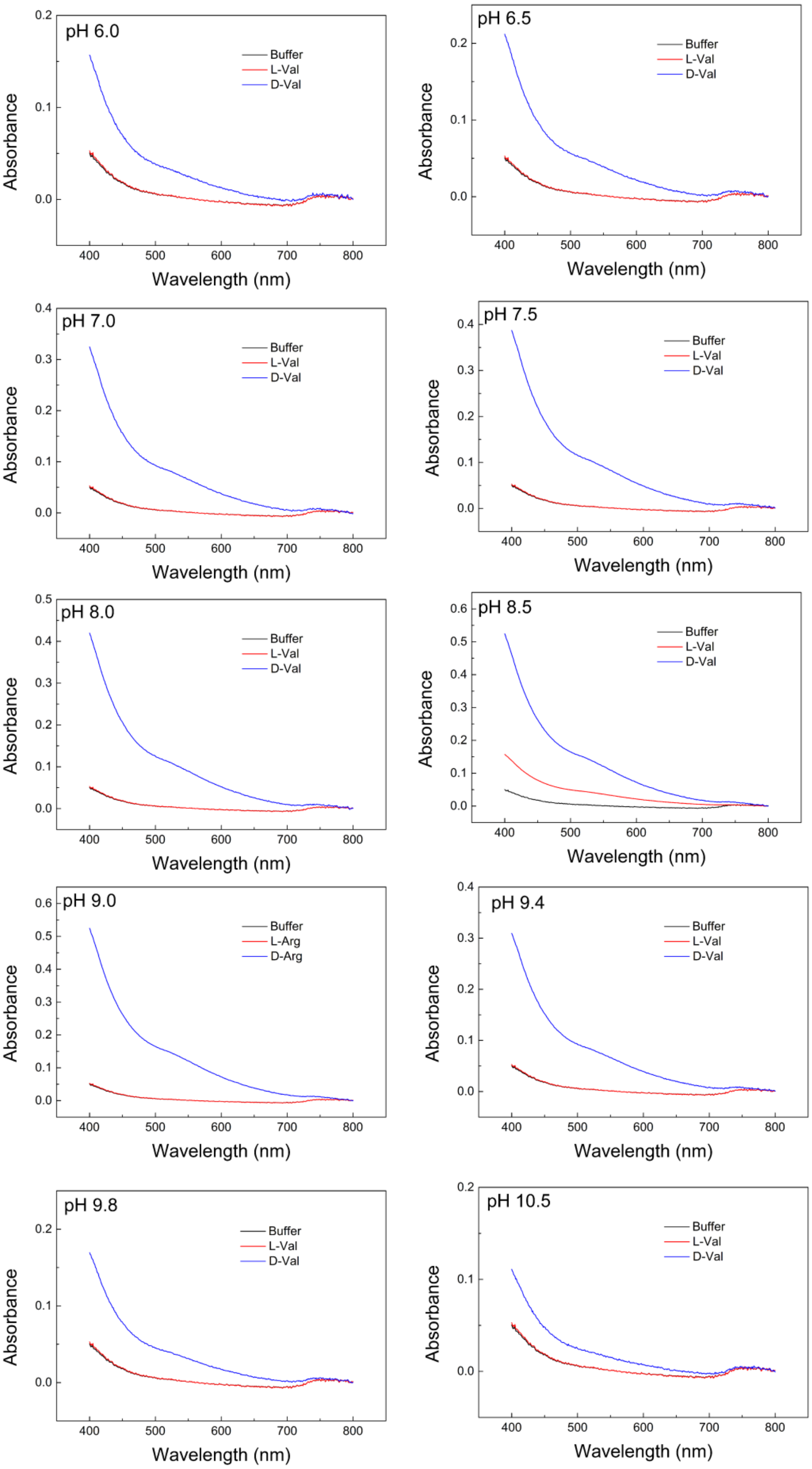

Figure S30 UV absorbance of UV-vis spectrum of the reaction product derived from DAAO and L-, D-Val, the concentration of L-Val and D-Val was 10 mM.

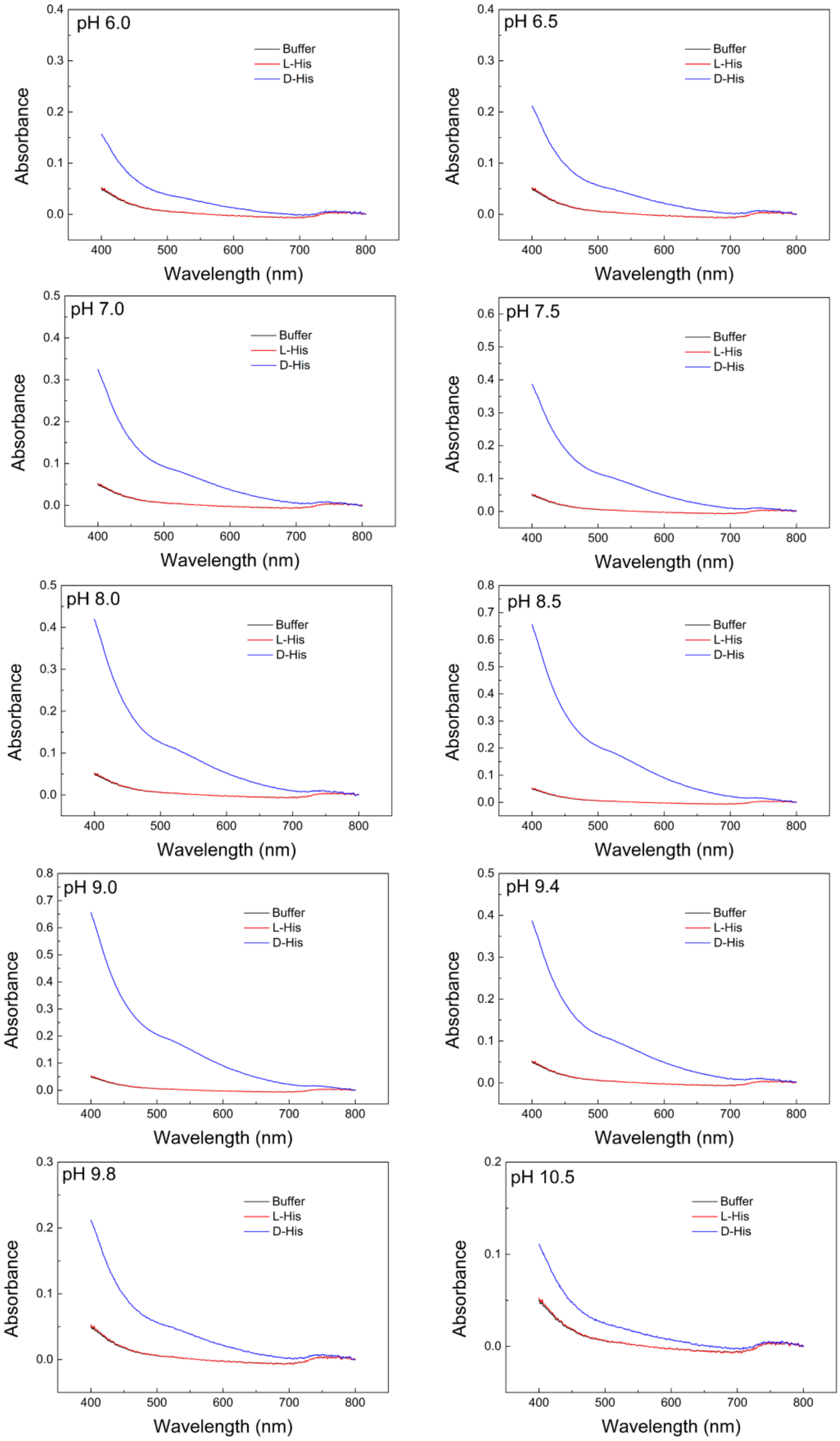

Figure S31 UV absorbance of UV-vis spectrum of the reaction product derived from DAAO and L-, D-His, the concentration of L-His and D-His was 10 mM.

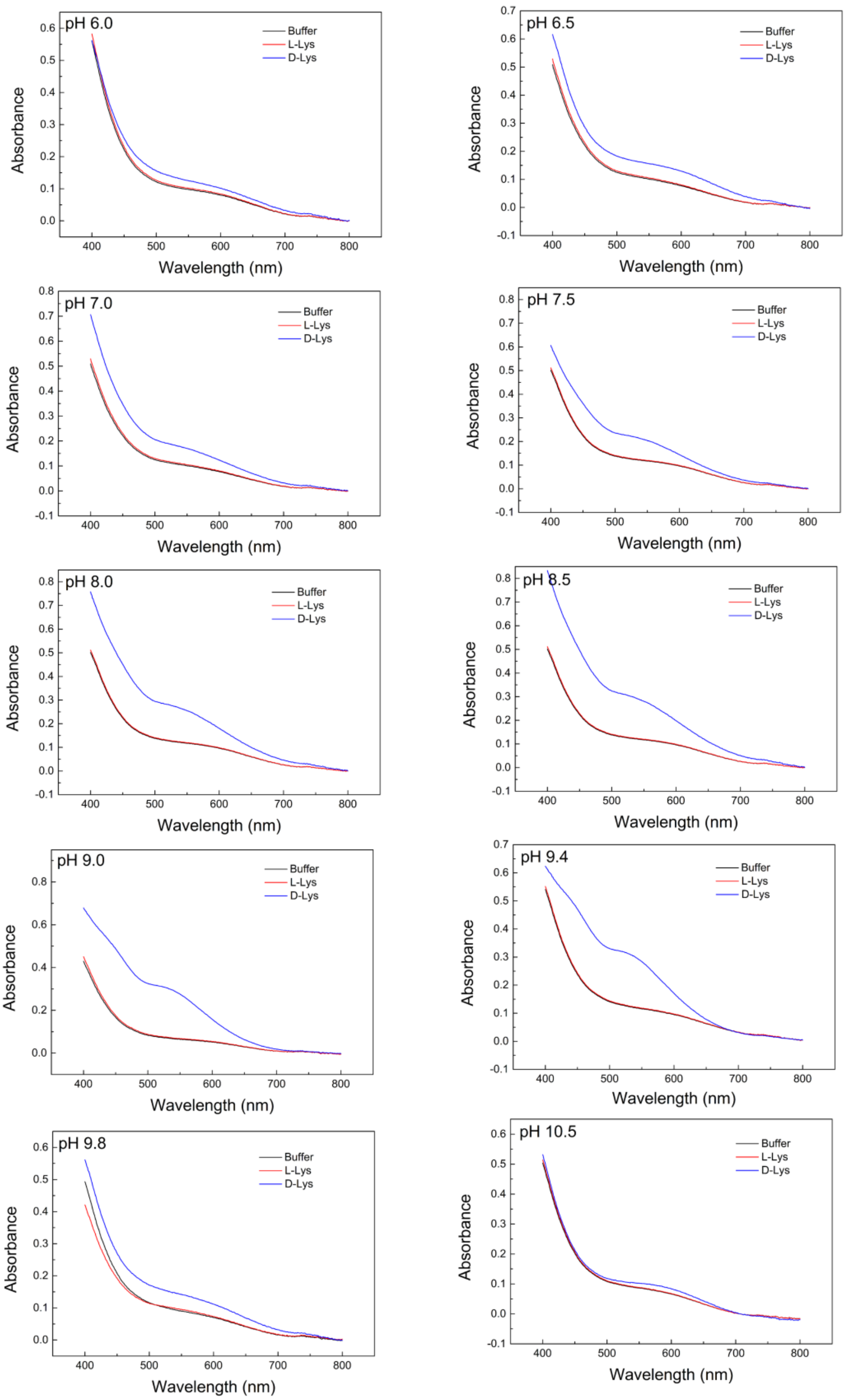

Figure S32 UV absorbance of UV-vis spectrum of the reaction product derived from DAAO and L-, D-Lys, the concentration of L-Lys and D-Lys was 10 mM.

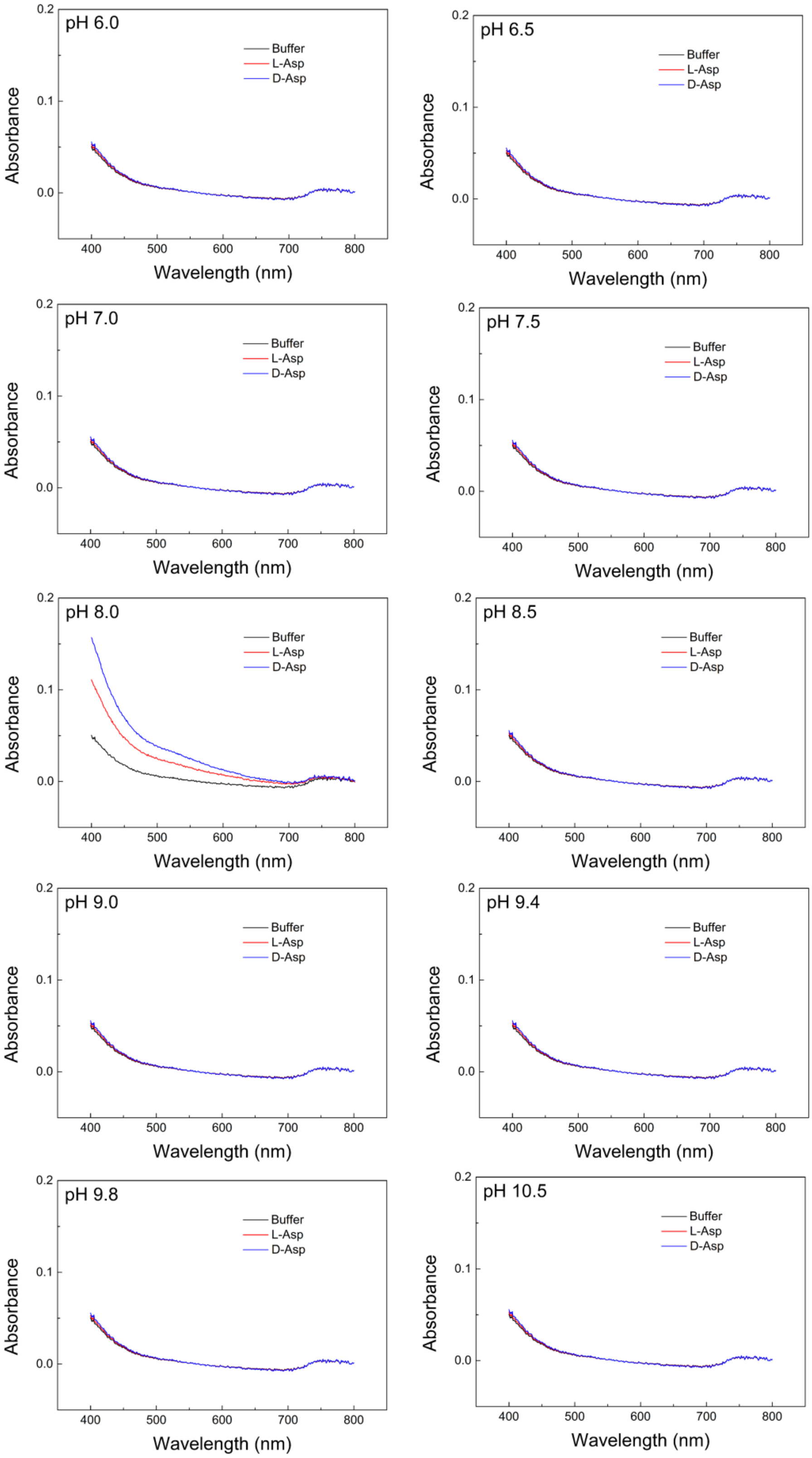

Figure S33 UV absorbance of UV-vis spectrum of the reaction product derived from DAAO and L-, D-Asp, the concentration of L-Asp and D-Asp was 1 mM.

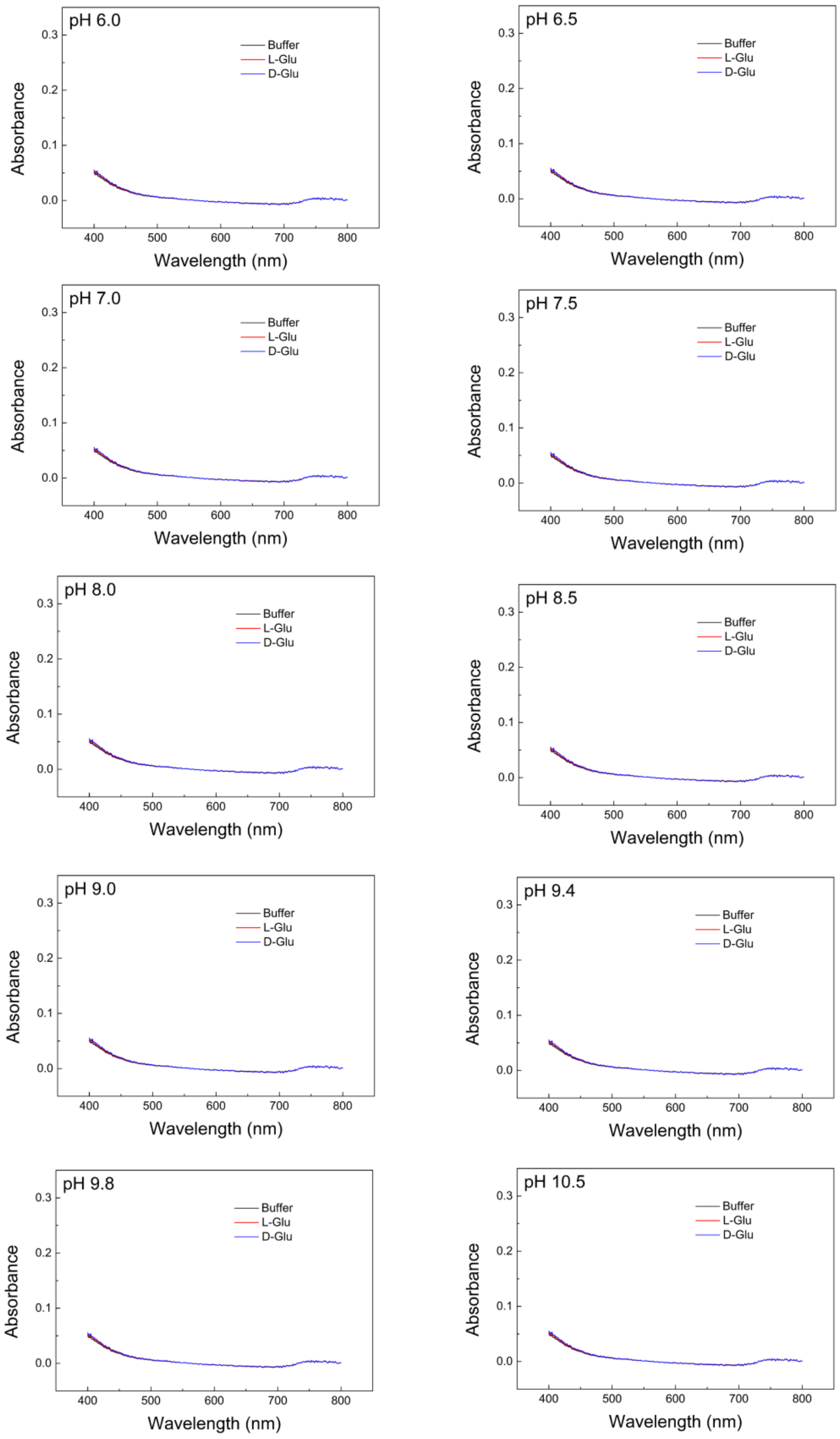

Figure S34 UV absorbance of UV-vis spectrum of the reaction product derived from DAAO and L-, D-Glu, the concentration of L-Glu and D-Glu was 1 mM.

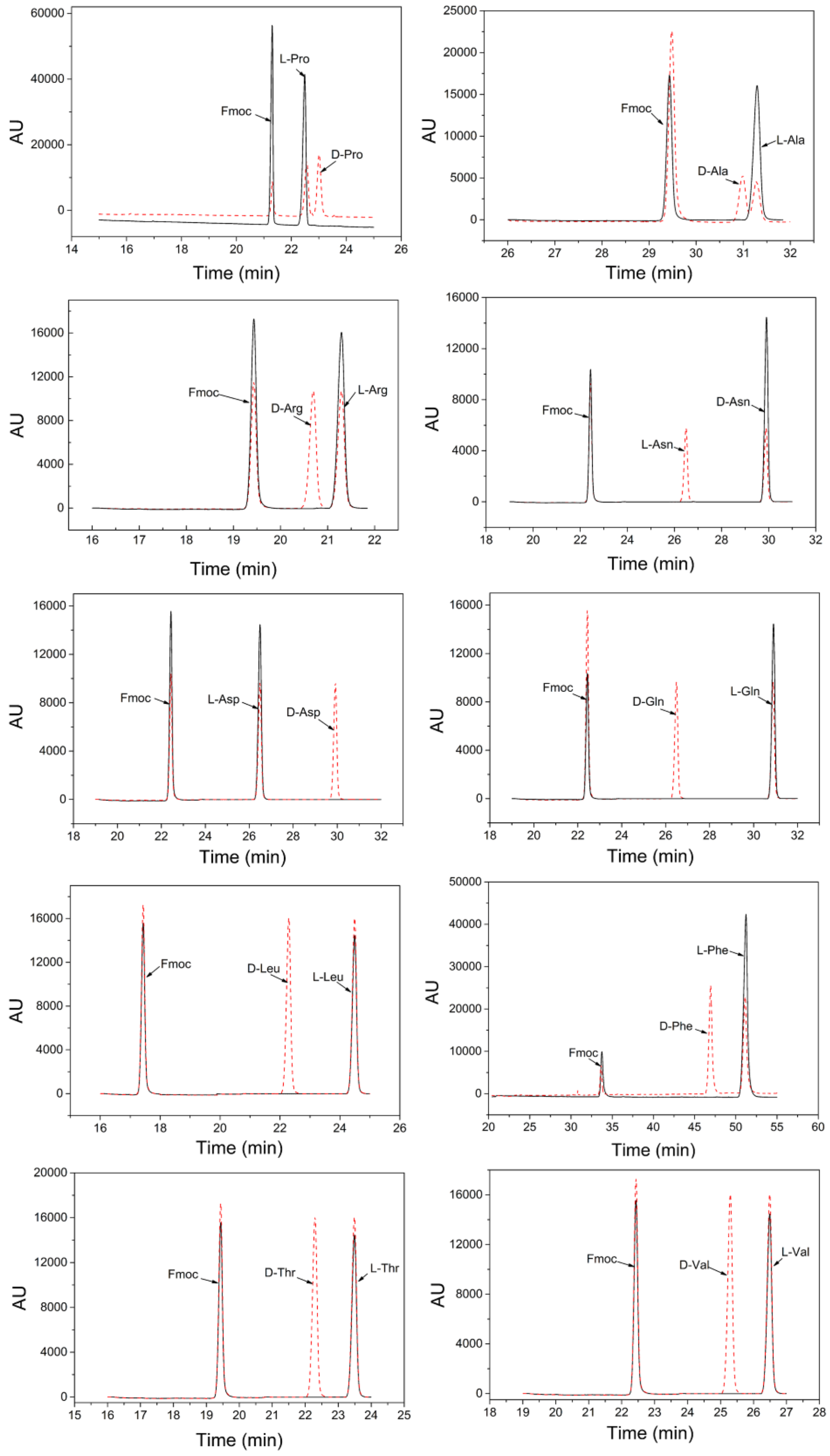

Figure S35 CE analysis of derived L-amino acid solutions. CE analysis of derived L-amino acid solution (solid line) after 30 min at 37 °C compared as mixtures of L, D-amino acid solution (dash line) after derivatization.

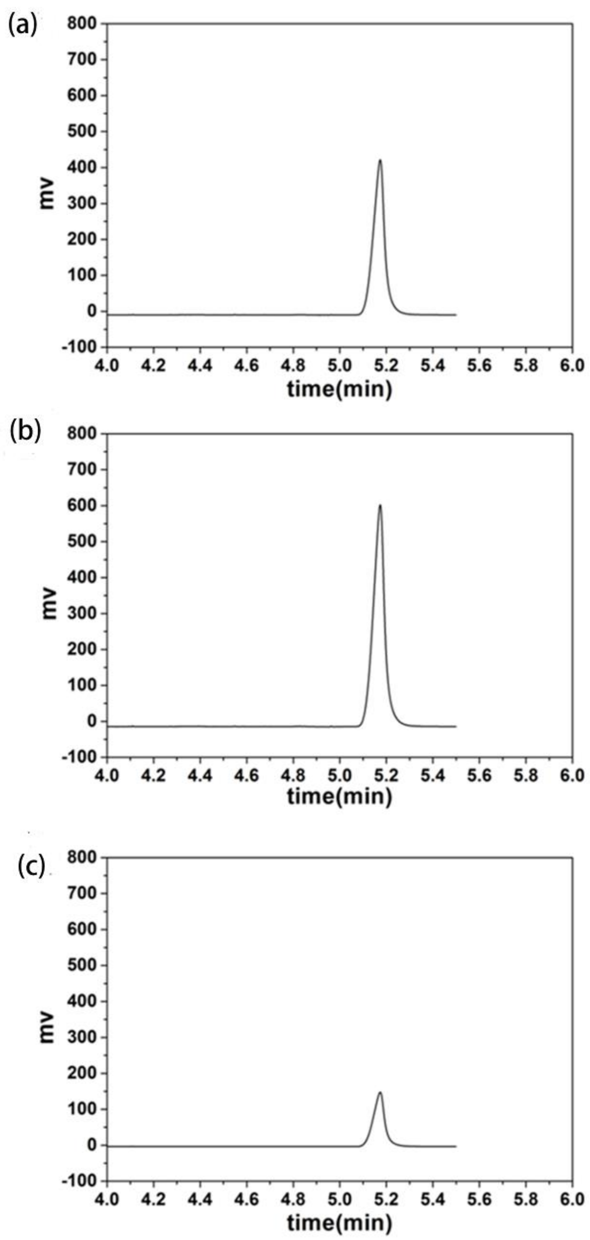

Figure S36 HPLC analysis of reaction product derived from Ala. (a) HPLC for 1.5 mM pyruvic acid (pH 9.8); (b) HPLC for reaction product derived from D-Ala (pH 9.8); (c) HPLC for reaction product derived from L-Ala (pH 9.8). The concentration of L-Ala and D-Ala was 10 mM, respectively.

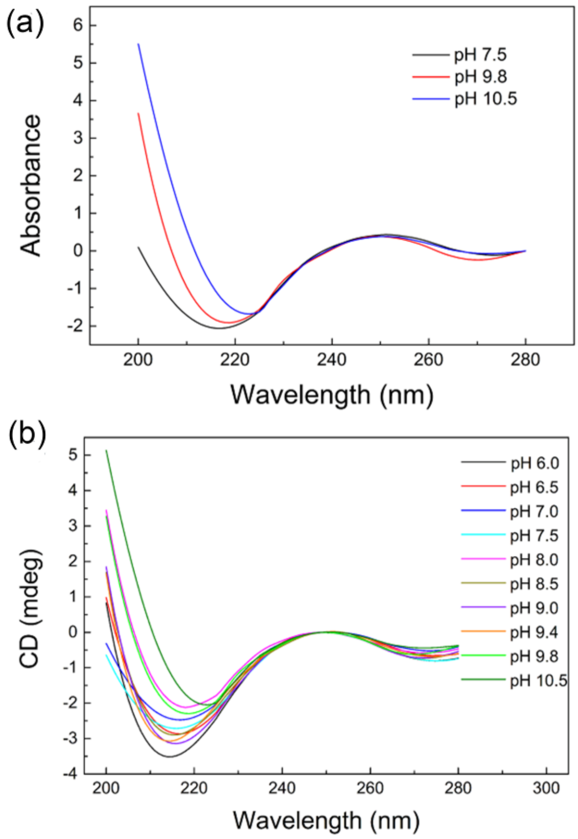

Figure S37 CD analysis of DAAO in presence of different pH values.

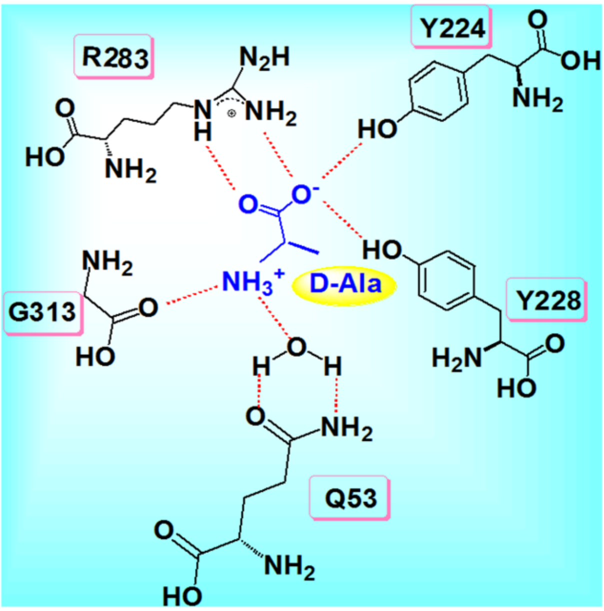

Figure S38 Schematic drawing of DAAO-D-Ala complex (PDB entry 1DDO). Hydrogen bonds between the ligand and active site residues were shown as dotted lines

Amino acids	Optimum pH	$k_{cat}(s^{-1})$	K_m(mM)	$k_{cat}/K_m(s^{-1}mM^{-1})$
L-Pro	8.0	6.7±0.41	27.0±2.0	0.25
L-Ala	9.8	9.4±0.39	43.1±3.6	0.22
l-Arg	6.5	1.8±0.19	39.0±4.1	0.046
L-Phe	9.0	9.1±0.11	45.2±5.8	0.202
L-Thr	8.0	2.6±0.53	27.3±3.2	0.096
L-Val	8.5	3.0±0.36	45.2±5.1	0.067
L-Leu	8.0	0.6±0.05	44.4±4.7	0.014
L-Asn	8.5	0.63±0.07	30.1±2.8	0.021
L-Gln	8.0	0.53±0.10	20.3±1.2	0.027

Table S1 Kinetic parameters of DAAO with L-amino acids as substrates.

Substrate	*Δ									
	pH 6.0	pH 6.5	pH 7.0	pH 7.5	pH 8.0	pH 8.5	pH 9.0	pH 9.4	pH 9.8	pH 10.5
Pro	-	0.65	2.39	6.19	16.8	4.27	3.58	3.22	2.9	-
Ala	-	-	-	-	-	-	-	-	18.18	-
Arg	-	11.68	-	-	-	-	-	-	-	-
Phe	-	-	-	-	-	-	1.7	-	-	-
Val	-	-	-	-	-	3.33	-	-	-	-
Met	-	-	-	-	-	-	-	-	-	-
Ser	-	-	-	-	-	-	-	-	-	-
Leu	-	-	-	-	-	-	-	-	0.49	-
Thr	-	-	-	-	-	-	-	5.38	-	-
Trp	-	-	-	-	-	-	-	-	-	-
His	-	-	-	-	-	-	-	-	-	-
Lys	-	-	-	-	-	-	-	-	-	-
Cys	-	-	-	-	-	-	-	-	-	-
Asn	-	-	-	-	-	0.58	-	-	-	-
Gln	-	-	-	-	0.46	-	-	-	-	-
Asp	-	-	-	-	58.3	-	-	-	-	-
Asn	-	-	-	-	-	-	-	-	-	-

Table S2 The comparison of activity of DAAO with L- and D-amino acid as substrate using indirect method with 2, 4-dinitrophenylhydrazine. *Δ = ($OD_{550}(P_{L\text{-}AA})$- OD_{550}(Control))/($OD_{550}(P_{D\text{-}AA})$- OD_{550}(Control))×100, where OD_{550} ($P_{L\text{-}AA}$) and OD_{550} ($P_{D\text{-}AA}$) are the absorbance of the product at 550 nm with L-amino acid and D-amino acid as substrate, respectively.
